# Novel pH-sensitive and biodegradable micelles for the combined delivery of doxorubicin and conferone to induce apoptosis in MDA-MB-231 breast cancer cell line†

**DOI:** 10.1039/d0ra03467c

**Published:** 2020-08-07

**Authors:** Akram Rahmani, Hassan Zavvar Mousavi, Roya Salehi, Ahmad Bagheri

**Affiliations:** Department of Applied Chemistry, Faculty of Chemistry, Semnan University Semnan Iran; Department of Chemistry, Faculty of Science, University of Guilan P.O. Box 41335-1914 Rasht Iran hzmousavi@semnan.ac.ir hzmousavi@guilan.ac.ir; Drug Applied Research Center, Tabriz University of Medical Sciences Tabriz Iran salehiro@tbzmed.ac.ir; Department of Medical Nanotechnology, School of Advanced Medical Sciences, Tabriz University of Medical Sciences Tabriz Iran

## Abstract

pH-sensitive micelles are desirable for co-drug delivery in cancer chemotherapy. Herein, a novel, very pH-sensitive and biodegradable citric acid grafted poly maleate-*block*-poly lactic-*co*-glycolic acid was synthesized and assembled as micelles *via* ultrasonication. The engineered homogeneous nanomicelles were used for the first time for doxorubicin and conferone combination chemotherapy in the MDA-MB-231 breast cancer cell line. The physicochemical properties of the micelles were investigated *via*^13^CNMR, ^1^HNMR, FTIR, CHNS, DSC, SEM, and DLS-zeta analysis, and the *in vitro* degradation of the synthetic copolymer was investigated to confirm its biodegradability. The critical micelle concentration (CMC) value of the micelles was determined using pyrene as a probe and a spectrofluorometer. The drug release process was studied in acidic and neutral pH. The anti-tumoral properties of the dual drug-loaded micelles were investigated *via* MTT assay, cell cycle, and apoptosis experiments. The apoptosis was confirmed by Annexin-V, qRT-PCR and western blotting. The particle size (51.9 nm), zeta potential (−6.57 mV) and CMC (1.793 μg mL^−1^) of the co-drug loaded micelles were in the acceptable range for electrostatic stability. The uptake of the co-drug loaded micelles in the MDA-MB-231 cell line and spheroids was 97% and 36.1%, respectively. The cell cycle and apoptosis tests revealed that the cells treated with the co-drug-loaded micelles showed the highest amount of apoptosis (95.35%) in comparison to the single drug-loaded micelles and free drugs. Reverse transcription PCR (RT-PCR) showed that the expression levels of the proapoptotic genes were significantly up-regulated in the presence of the co-drug loaded micelles *versus* the single-drug loaded micelles and free drugs. Western blotting revealed that the co-drug-loaded micelles promoted apoptosis *via* the caspase-dependent pathway. Our findings confirmed that the pH-responsive biodegradable micelles containing doxorubicin and conferone are novel and effective for combination chemotherapy and offer a promising strategy for future *in vivo* studies.

## Abstract 2

pH-sensitive micelles are desirable for co-drug delivery in cancer chemotherapy.

## Graphical abstract

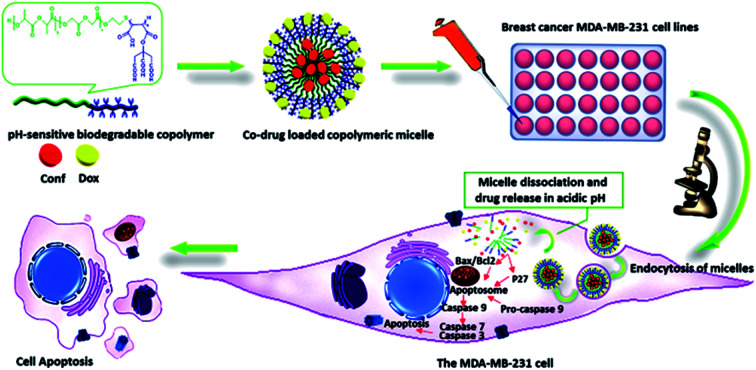

## Introduction

Multidrug resistance (MDR) and non-specific toxicity result in chemotherapy failure.^1–4^ Thus, combination therapy is used to overcome drug resistance in cancer patients.^2^ Although doxorubicin (Dox) is a potent and common chemotherapeutic agent, it possesses challenging side effects including cardiotoxicity, myelosuppression, nausea, vomiting, and loss of appetite.^5,6^ A common way to decrease the side effects of Dox is the utilization of adjuvant compounds, which reduce the therapeutic dosage and keeps or increases the therapeutic outcome.^7^ Different adjuvants are used in combination with Dox including cyclophosphamide,^8,9^ 5-fluorouracil,^7,10^ β-caryophyllene,^11^ thiocyanoacetamide,^12^ ifosfamide,^13^ furanodiene,^14^ quercetin,^15^ quinacrine,^16^ orange peel extract, naringin^17^ and conferone.^18^ Conferone is a natural and non-toxic compound, which is extracted from the fruits and roots of the self-growing *Ferula* species, which has significant anticancer properties, such as antiangiogenic effects, suppressing P-glycoprotein (P-gp)-mediated drug efflux, and increasing the cellular uptake and accumulation of Dox in cancer cells. However, the low water solubility of conferone decreases its bioavailability and limits its *in vivo* application.^19^ Therefore, in this study, we attempted to take advantage of the beneficial properties of conferone by overcoming its water insolubility *via* a new nano-formulation in order to decrease the effective dosage and side-effects of Dox.

PLGA is an FDA-approved biodegradable and biocompatible copolymer, which is a common carrier for nano-encapsulation and a suitable approach to overcome the insolubility of hydrophobic drugs in water.^20^

In recent decades, polymeric micelles, which spontaneously turn into a micellar core–shell structure in aqueous solutions, have received increasing attention. The major advantages of these polymeric micelles include their small size, tumour passive targeting through the enhanced permeability and retention effect (EPR), increasing the solubility of hydrophobic drugs in water, long circulation time, thermodynamic and kinetic stability, and possibility of functionalization and modification of their surface.^21–23^ Despite the advantages of polymeric micelles, the burst drug release by micelles and their lack of sensitivity to cancer cells are their main insufficiencies in the treatment of cancer.^24^ This problem is usually solved by increasing the sensitivity of the copolymeric micelles to the environment by using various chemical and physical stimuli. These mechanisms include high temperature, acidic pH, redox and ultrasonic or irradiation response, which are the significant differences between tumour cells and normal cells.^23,25,26^ Ramasamy *et al.* comprehensively described different types of sensitive copolymeric carriers and their synthetic preparation.^27–29^

Citrate is known as a ligand that can target CMT cancer cells *via* carrier-mediated transportation (CMT). The citrate transporter (NaCT) belongs to the SLC13 family and catalyzes the co-transportation of Na and citrate into the cell. It was shown that the expression profile of these transporters has been increased in many types of cancers.^30–33^ In this study, we employed citrate as a ligand to target CMT cancer cells *via* carrier-mediated transportation (CMT). Furthermore, to increase the solubility and pH sensitivity of the amphoteric PLGA-based copolymer, enhance the formation of micelles and drug-loading capacity of the micelles, and target cancer cells, a new biodegradable and biocompatible micelle was engineered using citric acid-grafted polymaleate-*block*-PLGA, which has four carboxylic acid groups per monomer unit in the hydrophilic portion of the copolymer. Citric acid was used to open the anhydride maleic polymer rings. The citrate portion has two roles in this study, where firstly, it acts as a ligand for targeting cancer cells, and secondly, as an active site for ionic interaction with Dox at physiological pH, which removes the ionic interaction at the pH (5.4) of tumor cell endosomes. To the best of our knowledge, this novel engineered micelle was used to investigate the co-delivery of doxorubicin–conferone to the MDA-MB-231 breast cancer cell line for the first time.

## Experimental

### Materials and instruments

Maleic anhydride (MA), citric acid (CA), l,d-lactide, glycolide, polyvinyl alcohol, 89 000–98 000 (PVA), azo-bis-isobutyronitrile (AIBN), tin(ii) octoate, propidium iodide (PI) and Tween®20 were purchased from Sigma-Aldrich (US). Sodium hydride (NaH, 60%, suspension in paraffin), 2-mercapto ethanol (ME) and all analytical grade solvents including toluene, *N*,*N*-dimethylformamide (DMF), diethyl ether (DEE), acetone, and dimethyl sulfoxide (DMSO) were provided from Merck Company (Germany). The MDA-MB-231 human breast cancer cell line was purchased from the Pasteur Institute. Thiazolyl blue tetrazolium bromide (MTT) was purchased from Alfa Aesar, Thermo Fisher Scientific (Heysham, UK). Penicillin-streptomycin 100× was obtained from Serana Europe GmbH (Germany). Trypsin–EDTA 0.25% (1×), fetal bovine serum (FBS) and Roswell Park Memorial Institute 1640 growth medium (RPMI 1640) were purchased from Gibco, Life Technologies Limited (UK). Conferone (Conf) was extracted from the roots of *Ferula flabelliloba* by Iranshahi *et al.*^34^ Doxorubicin (Ebedoxo®) was obtained by EBEWE Pharma (Austria). Ribonuclease A was purchased from Thermo Scientific (EU, Lithuania). ApoFlowEx® FITC Kit (apoptosis kit) was purchased from EXBIO Praha, a.s. (Czech Republic). TRIzol® Reagent was obtained from Life Technologies (USA).

The chemical structures of the synthetic copolymers (solid samples formed as KBr tablets) were determined *via* Fourier transform IR spectroscopy (FTIR, Bruker, Tensor 27, Germany) in the wavelength range of 400–4000 cm^−1^ and ^1^HNMR and ^13^CNMR spectroscopy on a Bruker 400 MHz spectrometer, Leipzig, Germany (copolymer solution in DMSO-d6). Elemental analysis (C, H, N, S, O%) of the copolymer was performed using a CHNS–O analyzer elemental combustion system (HromLab Costech elemental analyzer, elemental combustion system, ECS 4010, Germany). Differential scanning calorimetry (DSC) analysis was performed using a NETZSCH DSC 200 F3 Maia® (Germany) in a closed pan aluminum crucible, with pure nitrogen as the purge gas. In the first run, to eliminate the thermal history of the copolymer, the copolymer sample (6 mg) was heated to above its melting point (at a rate of 10 °C min^−1^), and then cooled to its lowest temperature (−90 °C) using liquid nitrogen. Subsequently, in the second run, the copolymer sample was heated again to 250 °C. The glass transition temperature (*T*_g_) was determined *via* DSC measurements. An ultrasonic cell crusher probe (SYCLON, SKL-500 II DN, Ningbo Haishu Sklon Electronic Instrument Co., Ltd., China) was used for the formation of micelles. The morphology and size of the micelles were investigated *via* field emission scanning electron microscopy (MIRA3-XMU TESCAN FESEM, Czech). The size of the micelles was determined by measuring the dimeter of at least 1322 micelles (prepared by SEM) using Image analysis software (Image-Pro Plus 4.5; Media Cybernetics, Silver Spring, MD). The particle size distribution and zeta potential of the samples were determined *via* dynamic laser scattering (DLS-Zetasizer Nano ZS90, Malvern Instruments, Malvern, UK). The Fluorescence emission study was performed using a spectrofluorometer (Jasco FP-750 spectrofluorometer, Japan). A UV-visible spectrophotometer (UV160-Shimadzo, Japan) was used to measure the drug content. A research fluorescence microscope (Nikon E1000M, Tokyo, Japan) equipped with a Planapo apochromatic objectives was also used (Nikon, Tokyo, Japan). An FACSCalibur flow cytometer (Becton Dickinson Immunocytometry Systems, San Jose, CA, USA) was used to measure the fluorescence intensity associated with Rhodamine B uptake inside the cells, cell cycle and apoptosis analysis. Total RNA was quantified using a NanoDrop (ND-1000, NanoDrop Technology, Australia). The RT-qPCR process was performed using a PeQlab® (UK) and Roche, LightCycler® 96 (USA).

### Copolymer synthesis

#### Synthesis of hydroxy terminated poly maleic anhydride

For the synthesis of the hydroxy-terminated polymaleic anhydride (P1: PMA–OH), firstly, maleic anhydride (MA, 3.93 g, 0.04 mol) was dissolved in dry toluene (60 mL) in a three-neck round-bottom flask under refluxing and nitrogen bubbling. After MA was completely dissolved, 2-mercapto ethanol (ME, 3.5 mL, 0.051 mol) was added with a syringe trough a septum-closed neck. N_2_ bubbling was continued for 15 min, and then, the temperature was increased to 110 °C and azo-bis-isobutyronitrile (AIBN, 1% mole of all monomers, 0.147 g, 0.0009 mol) dissolved in 5 mL dry toluene was added to the reaction mixture through septum injection. The reaction was continued for 20 h. After completion of the reaction, the yellowish viscose crude product was precipitated with acetone (solvent)/toluene (antisolvent) system. The product of this stage (P1) was freeze-dried for 48 h.

#### Functionalization of PMA–OH with citric acid grafting

Firstly, citric acid (CA, 1.715 g, 0.00816 mol) was dissolved in dry *N*,*N*-dimethyl formamide (DMF, 32 mL) in a two-necked round-bottom flask under N_2_ bubbling and stirring. After 10 min, solid sodium hydride (NaH, 0.51 g, 0.021 mol) was added to the solution gradually and mixed. CA activation was continued for 2 h. Then, the temperature was increased to 85 °C and PMA–OH (P1, 0.8 g, 0.00455 mol) solution in 8 mL dry DMF was added dropwise to activate the CA solution. The reaction was continued for 24 h. The white solid product was re-precipitated in diethyl ether (DEE) and then washed with acetone. The product of this stage, P2, (citric acid-grafted hydroxy-terminated polymaleate = CA-*g*-PMA–OH) was centrifuged and dried.

#### Post copolymerization of CA-*g*-PMA–OH with lactide and glycolide

CA-*g*-PMA–OH (P2, 0.7 g, 0.00181 mol), lactide (LA, 2.1 g, 0.0146 mol) and glycolide (GL, 1.05 g, 0.009 mol) were poured into a two-necked round-bottom flask under an N_2_ atmosphere at 160 °C to melt all the materials. Then, tin(ii) octoate (Sn (Oct)_2_, 3% w/w of all monomers, 0.076 mL) was added to the melted bulk as a catalyzer. The reaction was continued for 24 h. After cooling, the yellowish crude product was dissolved in dichloromethane (DCM) and precipitated in diethyl ether (DEE) three times. Then, the final product, citric acid-grafted poly maleate-*co*-PLGA, P, (CA-*g*-PMA-*co*-PLGA) was dried.

The chemical structure and physico-chemical properties of the synthesized copolymers were studied *via* FTIR, ^1^HNMR, ^13^CNMR, CHNS and DSC.

### Copolymer *in vitro* degradation test

For the degradation study, 10 mg of copolymer was dispersed in PBS with pH values of 7.4 and 5.5 in microtubes for different times (1, 6, 10, 16, 20, 26, 30, 35 and 40 day), separately, at 37 °C. After the predetermined time intervals, the samples were centrifuged (12 000 rpm, 25 min) to separate the polymer from PBS. After drying the copolymer *via* freeze-drying, it was weighed and analyzed using FTIR. Also, the pH of the supernatant PBS was determined with a pH-meter. Finally, the weight loss of the copolymer at different times and with different pH of the supernatant PBS was determined.

The weight loss (WL%) was calculated using following formula:^35^
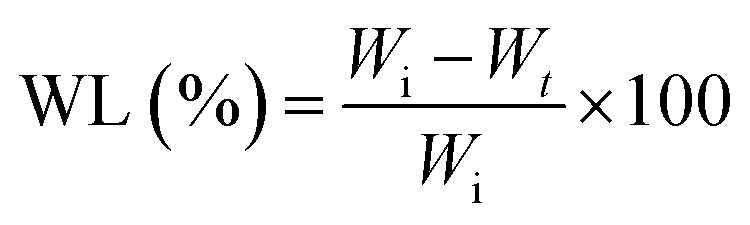
where *W*_i_ and *W*_*t*_ are the weight of the copolymer initially and at time, *t*, respectively.

### Critical micelle concentration (CMC) measurements

The CMC of the polymer was determined *via* the fluorescence technique using pyrene as a probe. The sample solutions were prepared by adding 1 mL of pyrene solution in acetone (1 mg pyrene in 10 mL acetone) to a series of flasks. As soon as the acetone evaporated completely, the polymer solutions in DMSO with various concentrations (0.01, 0.05, 0.5, 1, 2.5, 5, 10, 25, 50, 100, 250, 500, 1000 μg mL^−1^ final solution) were added to each of the flasks. Then the volume of each solution was adjusted to 20 mL with distilled water. All the prepared mixtures were sonicated using an ultrasound probe in the dark. The concentration of pyrene in the final solution was 5 × 10^−6^ mg mL^−1^ (0.005 μg mL^−1^). The flasks were maintained at 37 °C for about 18 h to equilibrate the pyrene partition between the water and micelles in a thermo-shaker. Subsequently, all the solutions were cooled to room temperature. Finally, the emission spectra of the micelle solutions were measured using a spectrofluorometer.

For the fluorescence emission spectra, the excitation wavelength was 334 nm and the emission wavelengths were 373 (*I*_1_) and 393 nm (*I*_3_).

### Preparation and characterization of blank and drug-loaded micelles

For the preparation of the blank polymeric micelles, 200 mg of polymer was dissolved in 6 mL DMSO, and then the polymer solution was added dropwise to 20 mL of polyvinyl alcohol (PVA) 1% w/v solution under sonication in an ice bath. The blank micelle solution was centrifuged using Amicon® centrifugal filters (Ultra-15, molecular weight cutoff of 100 kDa, Millipore, Darmstadt, Germany) at 4500 rpm for 10 min. The precipitant was freeze dried for the subsequent experiments.

Doxorubicin-loaded micelles were prepared *via* the dropwise addition of the polymer solution (200 mg in 6 mL DMSO) to PVA (20 mL, 1%) solution containing 20 mg doxorubicin under sonication in an ice bath. Upon the addition of the polymer solution, the pH of the PVA–Dox solution was regulated at 7.4 with dilute NaOH solution continuously. Finally, the Dox-loaded micelles were collected using Amicon® centrifugal filters at 4500 rpm for 10 min. The precipitant was freeze dried and kept at −20 °C.

Conferone loading was accomplished by adding the polymer and conferone solution (200 mg polymer and 20 mg conferone were dissolved in 6 mL DMSO) to 20 mL PVA 1% solution under sonication in an ice bath. The other steps were similar to that for Dox loading.

Similarly, for the simultaneous conferone and Dox loading, conferone and polymer solution (200 mg polymer and 10 mg conferone in 6 mL DMSO) was added gradually to PVA and Dox solution (10 mg Dox in 20 mL PVA 1%) under sonication in an ice bath. The pH of the solution was fixed at 7.4 with dilute NaOH solution with concurrent micellization. The subsequent stages were similar to that of the Dox loading process. Next, a UV-vis spectrophotometer was used to investigate the drug loading and release (*λ*_max_,_Dox_ = 480 nm and *λ*_max,conferone_ = 324 nm).

The drug encapsulation efficiency (DEE%) was calculated using the following equation:^36^
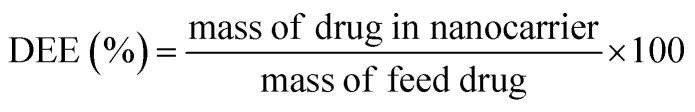


### Micelle characterization

The morphology and size of the micelles were investigated *via* SEM. The blank and co-drug-loaded micelles were analyzed *via* FTIR spectroscopy and zeta potential measurements to confirm the drug loading in the micelles. Also, the diameter and zeta potential of the micelles were evaluated *via* DLS zeta measurements.

### *In vitro* drug release

The single- and co-drug-loaded micelles (PD, PC, and P2D) (2 mg) were dispersed in 2 mL sink solution (99% PBS, 0.5% DMSO and 0.5% Tween® 20) with pH values of 5.5 and 7.4 at 37 °C, then the microtubes were situate in a shaker-incubator for 17 days. After predetermined times (1, 2, 3, 24, 48, 72, 96, 168, 336, and 408 h), the microtubes were centrifuged at 8000 rpm for 25 min and the supernatant solutions (2 mL) were collected and replaced with the same amount of fresh sink solution. The percentage drug release was calculated using the following equation:^37^
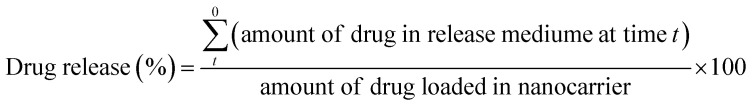


### Cell culturing

The MDA-MB-231 breast cancer cell line was cultured in a T25 culture flask. The cells were cultured in RPMI-1640 medium containing 10% fetal bovine serum (FBS), 1% penicillin (50 IU mL^−1^) and streptomycin (50 μg mL^−1^) at 37 °C in a humidified incubator supplied with 5% CO_2_.

### Intracellular uptake study

Flowcytometric analysis was used to study the cellular uptake of the blank and co-drug-loaded polymeric micelles containing Rhodamine B in the MDA-MB-231 cell line. To prepare the Rhodamine B-labeled blank micelles (RB-P), 10 mg copolymer and 0.1 mg Rhodamine B (RB) were dissolved in 1 mL DMSO. Then this solution was added dropwise to 4 mL PVA 1% w/v solution, under probe sonication in the dark in an ice bath. Then, the RB-labeled micelles (RB-P) were centrifuged. The micelle precipitant was washed with distilled water and centrifuged three times to eliminate the unloaded Rhodamine B. Finally, the RB-P micelles were dispersed in 1 mL distilled water and frozen for later use.

The Rhodamine B-labeled co-drug-loaded micelles (RB-P2D) were prepared similarly. In this case, 0.5 mg conferone, 0.1 mg Rhodamine B (RB) and 10 mg copolymer were dissolved in DMSO and 0.5 mg doxorubicin was added to 1% PVA solution. The other steps were performed according to the previous process. The pH of the RB-P and RB-P2D micellar solutions was adjusted to 7.4.

In the next step, the MDA-MB-231 cells were seeded in 6-well plates at a density of 5 × 10^5^ per well and incubated for 48 h. The cells without any treatment were considered as the negative control. The cells were treated with RB-P and RB-P2D micelles. After 0.5, 1.5, and 3 h, the cells were trypsinized, washed with PBS and examined using an FACSCalibur flow cytometer to measure the fluorescent intensity of RB uptake inside the cells.

Also, the intracellular uptake of the RB-P2D-loaded micelles was further investigated using a research fluorescence microscope (Nikon E1000M, Tokyo, Japan) equipped with a Planapo apochromatic objectives. The MDA-MB-231 cells were seeded on a slide chamber. After 48 h, the cells were treated with RB-P2D micelles. The slide chambers were incubated for 1 h, and then the cells were washed with PBS. Finally, images of the RB-P2D micelle uptake were taken using the abovementioned fluorescence microscope.

### Study of the uptake of blank micelles and co-drug-loaded micelles into MDA-MB-231 spheroids

#### MDA-MB-231 spheroid culture and growth using hanging-drop method

This experiment was carried out based on the method reported by Tchoryk *et al.*^38^ with a slight modification. Monolayer-grown MDA-MB-231 cells were harvested with trypsin and centrifuged. After counting, the diluted cells (20 000 cells per drop) were seeded as hanging drops on a plate cap containing PBS for spheroid formation, and then incubated for 72 h at 37 °C under 5% CO_2_. Then, for growth and compaction, the spheroids were placed in round-bottom glass tubes separately (containing 2 mL complete medium with 10% FBS), and incubated in a shaker-incubator (37 °C, 5% CO_2_) for 3 days.

#### Study of uptake and penetration of RB-labelled blank- and co-drug-loaded micelles in spheroids by flow cytometry

The compact spheroids (about 1000 μm diameter) were transferred into a six-well (one spheroid per well containing 2 mL complete medium with 10% FBS) and were treated with RB-P (P: 5 and 10 μg mL^−1^) and RB-P2D micelles (P2D: 0.5 and 1 μg mL^−1^ containing 5 and 10 μg mL^−1^P, respectively), for 24 h. Then, the media of all the wells were discarded, and the spheroids were stained with Hoechst (1 mL, 0.1 μM per well) and incubated for 4 h. Subsequently, the spheroids were washed with PBS, harvested with trypsin (for complete dissociation of spheroids, the cells suspension was pipetted gently) and then centrifuged. Finally, the cells were washed with PBS twice and analyzed using an FACSCalibur flow cytometer. The untreated cells, and Hoechst-stained cells (were prepared in the same way) were used for the threshold determination.

### *In vitro* cytotoxicity assay and combination index analysis

After reaching 70% confluency, the cultured cells were trypsinized and centrifuged at 1000 rpm for 5 min at room temperature. Subsequently, the cells were seeded in 96-well plates (at a cell density of 10 000 cells per well) in 200 μL complete RPMI medium, and incubated for 48 h. The cytotoxicity of the free single drug (Conf and Dox), free Dox–Conf combination (2D), blank micelles (P), single- (P-Dox: PD and P-Conf: PC) and co-drug-loaded (P2D) micelles were investigated against MDA-MB-231 cells using the MTT assay. After discharging the old medium from the plates, the cells were treated with various concentrations of Dox, Conf, 2D, PD, PC, P2D (0.058, 0.117, 0.234, 0.468, 0.937, 1.875 and 3.75 μg mL^−1^) and blank micelles (31.25, 62.5, 125, and 250 μg mL^−1^) for 4 h. Then the medium containing the remaining micelles and drugs was removed and replaced with fresh complete medium and incubated for 48 h. All the concentrations were measured in triplicate. After evacuating the medium from the wells, 50 μL of MTT solution (2 mg mL^−1^) in PBS (pH 7.4) and 150 μL complete medium were added to all the wells in the dark and incubated for a further 4 h at 37 °C. Then, the medium containing MTT was carefully removed from the wells and the formazan crystals, obtained from the living cells reacting with MTT, were dissolved in 200 μL of DMSO. Finally, after shaking for 10 min in the dark, the absorbance of the wells was measured at 492/630 nm using a microplate ELISA reader. Also, the cytotoxicity of the free Dox and free Conf (0.019, 0.39, 0.781, 1.562, and 3.125 μg mL^−1^) on normal renal cells (HEK 293) was investigated using the MTT method under the same conditions. The IC_50_ doses of all the formulations were calculated using the Prism software. Also, the combination index (CI) of the formulations was calculated using the CompuSyn v.1 software. If the CI value is 1, it shows an additive effect. On the other hand, CI < 1 and CI > 1 values reveal synergism and antagonism, respectively.

### Cell cycle study

MDA-MB-231 cells were seeded in 6-well culture plates at a density of 5 × 10^5^ per well. After 48 h incubation (37 °C, 5% CO_2_), the cells were treated with the free drugs and blank, single and co-drug-loaded micelles at their IC_50_ doses for 4 h. Then, the medium containing the remaining micelles and drugs was removed and replaced with fresh complete medium and incubated for 48 h. The cells without any treatment were considered as the controls. Then, the supernatant of the cells was collected in centrifuge tubes separately and the cells were rinsed with PBS, trypsinized and collected in the related tubes. All the cell-containing tubes were centrifuged, and their supernatants were removed. The cells precipitant was dispersed with 700 μL fresh and cold PBS and were centrifuged again. After the supernatant was removed, 300 μL PBS was added to every cell-containing tube and mixed. Subsequently, 700 μL cold ethanol (70%, 4 °C) was added to each of the tubes to fix the cells. The fixed-cell-containing tubes were placed in 4 °C for 72 h in the dark. Then, all the tubes were centrifuged, and their supernatants were removed. 300 μL PBS was poured into each tube, and subsequently, 10 μL of ribonuclease A was added to the tubes. After 45 min incubation at 37 °C, the cells were vortexed and stained with 10 μL propidium iodide (PI). Then, the tubes were placed in the dark for 10 min. Finally, the population frequencies in the different cell cycle phases were estimated with an FACSCalibur flow cytometer.

### Apoptosis assay

Apoptosis in the cells was investigated using the Annexin V-FITC/PI apoptosis detection kit. The cells were seeded in 6-well culture plates at a density of 1 × 10^5^ per well. After 48 h incubation (37 °C, 5% CO_2_), the cells were treated with free drugs, blank micelles, single- and co-drug-loaded micelles at their IC_50_ doses for 4 h. Then the medium containing the remaining micelles and drugs was removed and replaced with fresh complete medium and incubated for 48 h. Then, the supernatants of the cultured cells were collected in tubes separately, and then cells were washed with PBS and added to the corresponding tubes. Afterwards, the cells were trypsinized, centrifuged, and their supernatants were removed, and then the cells were washed with PBS twice. After centrifuging, the supernatants were discarded, and according to the manufacturer's protocol of the Annexin V staining kit (Exbio), the cells were washed with annexin binding buffer (BB). Subsequently, the cells were resuspended in 100 μL binding buffer again and then 5 μL of Annexin V-FITC (fluorochrome-conjugated Annexin V) and 5 μL of propidium iodide (PI) were added to all the samples and mixed gently. The cells were incubated for 15 min in the dark at room temperature. Subsequently, the cells were centrifuged and resuspended in 100 μL of binding buffer. Finally, the samples were immediately analyzed using an FACSCalibur flow cytometer. The cells without staining were also analysed as the auto-fluorescence reference.

### RNA isolation and cDNA synthesis

The MDA-MB-231 cells were treated with P, P2D, PD, PC, 2D, Dox and Conf at sub-IC_50_ doses for 4 h. Then, the medium containing the remaining micelles and drugs was removed and replaced with fresh complete medium and incubated for 48 h. The cells were washed with PBS twice and then harvested. Non-treated cells were used as the control. Then, the total RNA was isolated from the treated MDA-MB-231 cell line using the TRIzol® method. Briefly, the cells were precipitated by centrifugation at 500*g* at 4 °C, the plates were subjected to cell lysis using 750 μL of RiboEx, and then 200 μL chloroform was added to the lysate and incubated for 2 min at room temperature and centrifuged at 12 000*g* for 20 min (4 °C). The aqueous phase was collected, and one volume of isopropyl alcohol was added for the precipitation of total RNA at 12 000*g* for 20 min (4 °C). The RNA plate was washed with 75% ethanol alcohol and dissolved in DEPC-treated water and subsequently quantified using a NanoDrop. Complementary DNA (cDNA) synthesis was performed using Revert Aid Reverse Transcriptase (cat. no. EP0441, Thermo Scientific, Lithuania) according to the manufacturer's instructions.

### Quantitative PCR

qPCR was performed to detect the apoptosis pathway according to the following PCR program: initial denaturation at 95 °C for 15 min, 45 cycles of denaturation at 95 °C for 15 s, and annealing/extension at 60 °C for 50 s. The qPCR mixture contained 5 μL 2× SYBR Green Master Mix (RealQ Plus 2× Master Mix Green, Ampliqon, Denmark), 2 μL cDNA, 0.5 μL of 5 pmol μL^−1^ primer pair mix (Eurofin, Germany), and 3 μL H_2_O. The sequences of the primers are listed in Table 1. The glyceraldehyde-3-phosphate dehydrogenase (GAPDH) gene was used as the reference gene and the −ΔΔ*C*_t_ method was used to calculate the fold changes.

**Table 1:** Sequences of the primers

Gene	Forward primer (5′–3′)	Reverse primer (5′–3′)
CASPASE-3	GAAATTGTGGAATTGATGCGTGA	CTACAACGATCCCCTCTGAAAAA
CASPASE-6	ATGGCGAAGGCAATCACATTT	GTGCTGGTTTCCCCGACAT
CASPASE-7	AGGGACCGAGCTTGATGATG	CACTGGGATCTTGTATCGAGGA
CASPASE-8	GATCAAGCCCCACGATGAC	CCTGTCCATCAGTGCCATAG
CASPASE-9	CTTCGTTTCTGCGAACTAACAGG	GCACCACTGGGGTAAGGTTT
CASPASE-10	AGAAACCTGCTCTACGAACTGT	GGGAAGCGAGTCTTTCAGAAG
CASPASE-12	TGTTACAAAGGCTCATGTGGAAA	GGGTCAGTATATTTGGGGTCTCA
Bax	TTCTGACGGCAACTTCAACT	CAGCCCATGATGGTTCTGAT
Bcl-2	GGGAATCGATCTGGAAATCCTC	GGCAACGATCCCATCAATCT
GAPDH	ACAACTTTGGTATCGTGGAAGG	GCCATCACGCCACAGTTTC

### Western blotting

MDA-MB-231 cells were treated for 48 h with the P2D nano-formulation in sub-IC_50_ doses, and the cells were collected for lysing using the following protocol: RIPA buffer [500 μL of Tris–HCL (pH = 8), 1 tablet of protease inhibitor cocktail, 0.003 g EDTA, 0.08 g NaCl, 0.025 g sodium deoxycholate, 0.01 g SDS and 10 μL of Triton NP40 (1%)] at 4 °C. Finally, the cells were collected by centrifugation at 12 000 rpm for 10 min at 4 °C. The cell supernatant was analysed using the Bradford assay (Bio-Rad protein assay, Bio-Rad Laboratories, USA) and a spectrophotometer (Bibby Scientific Ltd, Beacon Rd, UK) for protein determination. The target protein fragments were obtained from sodium dodecyl sulfate polyacrylamide gel electrophoresis (SDS-PAGE) and transferred to a polyvinylidene difluoride (PVDF) membrane and blocked with TBST buffer containing 5% (w/v) skim milk (0.1% v/v Tween®20-tris buffered saline: TBST). The blocked PVDF membranes containing the target proteins were incubated with specific primary antibodies (Bax (B-9) mouse monoclonal antibody (Santa Cruz) (1 : 1000), Bcl-2 sc-492 (Santa Cruz) rabbit monoclonal antibody (1 : 1000), caspase-7 (C7) (Cell Signaling) rabbit polyclonal antibody (1 : 1000), caspase-3 (Cell Signaling) rabbit monoclonal antibody (1 : 1000), caspase-9 (Cell Signaling) rabbit polyclonal antibody (1 : 1000), GAPDH (Santa Cruz) mouse monoclonal antibody (1 : 1000) and P27 (Elabscience) rabbit polyclonal antibody (1 : 400) diluted with blocking buffer) overnight at 4 °C. The membrane was washed with TBST and incubated with secondary antibodies (m-IgGκ BP-HRP (Santa Cruz) for Bax (B-9), Bcl-2 and GAPDH, and mouse anti-rabbit IG-HRP (Santa Cruz) was used for caspase-3, 7, 9 and P27, which were diluted using blocking buffer (1 : 1000)) for 1 h at room temperature. The target protein bands were visualized using an enhanced chemiluminescence detection kit (Thermo Fisher Scientific, Breda, the Netherlands) and an Amersham® Imager 600 system (GE Healthcare Life Sciences, Eindhoven, the Netherlands). Finally, the western blotting results were normalized by the expression of GAPDH as the loading control. All protein bands were quantified using the Image J software (v. 1.52n).

### Statistical analyses

The results were analyzed in duplicate or triplicate and expressed as mean ± standard deviation (SD) using Microsoft Excel 2019 or GraphPad Prism software (v. 8). Statistical analyses were performed using the Student's *t*-test and ANOVA for two groups and multiples comparison, respectively. A *p* value less than 0.05 was considered to be statistically significant.

## Results and discussion

**Fig. 1:**
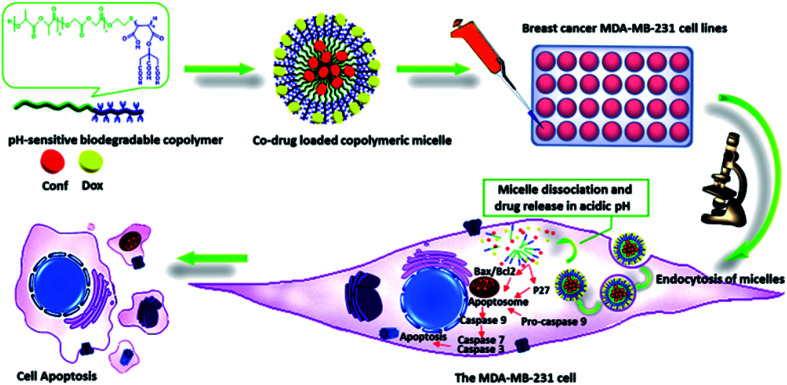
Schematic overview of the procedure.

A general schematic of the procedure in this study is shown in Fig. 1.

### Copolymer design and synthesis

**Fig. 2:**
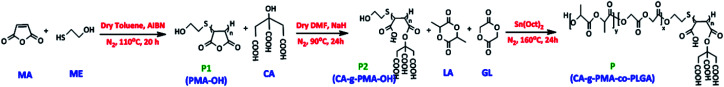
Steps in the synthetic process. Step 1: synthesis of hydroxy-terminated polymaleic anhydride (P1: PMA–OH), Step 2: functionalization of PMA–OH with citric acid (P2: CA-*g*-PMA–OH), and Step 3: post co-polymerization of CA-*g*-PMA–OH with lactide and glycolide (P: CA-*g*-PMA-*co*-PLGA).

The pH-sensitive biodegradable *block*-copolymer was synthesized using a 3-step synthetic route (Fig. 2). The synthesis of the hydroxyl-terminated polymaleic anhydride was performed *via* thiol–ene addition between 2-mercaptoethanol (ME) and maleic anhydride (MA) in the presence of AIBN as a radical initiator. In this mechanism, the radical initiator caused the production of ME radicals (HO–CH_2_–CH_2_–S˙), which attacked the maleic anhydride ene-bonds and initiated the radical polymerization of maleic anhydride. Thus, the product in this stage (P1) had an –OH end, which was essential for the copolymerization of lactide and glycolide in the final stage. The efficiency percentage of this stage was 44.25% (3.5 g).

To create a pH-sensitive copolymer and increase the water solubility of the copolymer and Dox–copolymeric micelle electrostatic interaction, the hydroxyl group (–OH) of citric acid (CA) was activated with NaH and deprotonated, which reacted with P1 (PMA–OH) *via* the ring-opening esterification of the maleic anhydride rings. P2 (CA-*g*-PMA–OH) was obtained in a yield of 61.63% (1.55 g) and the pH of this product was 4.5 due to the four carboxylic acids per unit, corresponding to maleic and citric acid.

Finally, the PLGA tail, which was expected to act as the hydrophobic core of the micelle and increase the biodegradability and biocompatibility of copolymer, was added to P2*via* the melt bulk ring-opening process to produce P (CA-*g*-PMA-*co*-PLGA). In this method, the –OH end group of P2 acted as a ring-opening agent, and in the presence of the Sn(Oct)_2_ catalyst, caused the ring-opening random copolymerization of lactide and glycolide. The yield of the final product (P) was 65.97% (2.54 g).

### Copolymer characterization

#### Fourier transform infrared spectroscopy (FTIR)

The chemical groups present in P1 (PMA–OH), P2 (CA-*g*-PMA–OH), and P (CA-*g*-PMA-*co*-PLGA) were studied *via* FTIR spectroscopy (Fig. 2-S†).

As shown in Fig. 2-S-P1,† the peaks in the range of 1100–1500 cm^−1^ are related to the (C–O–C) stretching of the anhydride rings and (C–O) group of 2-meracapto ethanol. The (C=O) symmetric and asymmetric stretching band of the anhydride groups appeared at 1548–1992 cm^−1^. The peaks at 2700–3000 cm^−1^ and very broad peaks at 3000–3605 cm^−1^ are attributed to the (CH and CH_2_) and (–OH) groups of maleic anhydride (CH) and mercapto ethanol (S–CH_2_–CH_2_–OH) sections of the P1 polymer, respectively. The presence of anhydride peaks and disappearance of the maleic anhydride alkene peaks suggest that the maleic anhydride double bonds were converted to single bonds without maleic anhydride ring opening.

In the case of Fig. 2-S-P2,† the peaks at 1053–1398 cm^−1^ are attributed to the (C–O) stretching of the ester and carboxylic acids groups. The signals of the stretching of the carbonyl groups (C=O) of the esters and carboxylic acid units in the citric acid and polymaleate sections were observed at 1579–1720 cm^−1^. The peaks at 2967 and 3496 cm^−1^ are assigned to the (CH and CH_2_) and (–OH) groups, respectively. The presence of carboxylic acid groups and disappearance of the anhydride peaks show that this stage of the synthesis was successfully performed.

In Fig. 2-S-P,† the (C–O) stretching band of the ester and carboxylic acid groups appear at 1078–1186 cm^−1^ and 1307–1398 cm^−1^, respectively. The band at 1762 cm^−1^ corresponds to the stretching of (C=O) groups of the ester and carboxylic acid units. The peaks at 2882–2997 cm^−1^ are related to the (CH, CH_2_, and CH_3_) groups and the peaks at 3300–3500 cm^−1^ correspond to the (–OH) groups of the PLGA end group and carboxylic acid units.

#### ^1^HNMR and ^13^CNMR analyses

**Fig. 3:**
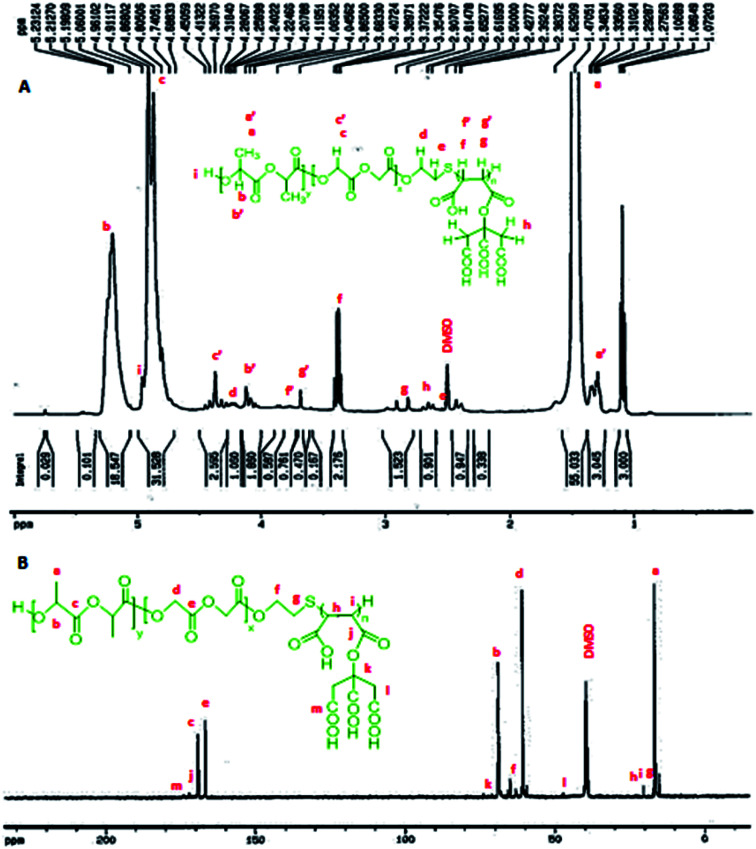
(A) ^1^HNMR spectrum of copolymer (P), where a′, b′ and c′ are the peaks related to the end groups, and f′ and g′ correspond to the maleate group bonded to –S. (B) ^13^CNMR spectrum of P.

The chemical structures of P1, P2 and P were determined *via*^1^HNMR and ^13^CNMR spectroscopy. As shown in Fig. 3-S,† in the ^1^HNMR spectrum of P1 (PMA–OH), the peaks related to the polymaleic anhydride units appeared at: *δ* = 2.886 ppm, 2H, PMA end (–C(H) (CO)–O–(CO) C**H̲**_2_) group; *δ* = 3.356 ppm, 2H, PMA backbone (–C(**H̲**) (CO)–O–(CO) C(**H̲**)–); *δ* = 3.466 ppm, 1H, (–S–C(H) (CO)–O–(CO) C(**H̲**)–PMA); *δ* = 3.584 ppm, 1H, (–S–C(**H̲**) (CO)–O–(CO) C(H)–PMA). The mercapto ethanol linkage signals were located at: *δ* = 2.70, 2.72 ppm, 2H, (PMA–S–C**H̲**_2_–C(H)–OH); *δ* = 4.07, 4.11 ppm, 2H, (PMA–S–CH_2_–C(**H̲**_2_)–OH); *δ* = 4.7–4.9 ppm, 2H, (PMA–S–C**H̲**_2_–C(H_2_)–OH).

In Fig. 3-S,† in the ^13^CNMR spectrum of the P1, the peaks of PMA appear at: *δ* = 29 ppm, PMA end (–C(H) (CO)–O–(CO) **C̲**H_2_) group; *δ* = 40.41 ppm, (–**C̲**(H) (CO)–O–(CO) **C̲**(H)–); *δ* = 59.2 ppm, (–S–**C̲**(H) (CO)–O–(CO) C(H)–PMA); *δ* = 166.5–174 ppm, (–C(H) (**C̲**O)–O–(**C̲**O) C(H)–). The signals of the mercapto group are present at: *δ* = 30 ppm, (PMA–S–**C̲**H_2_–C(H_2_)–OH); *δ* = 60–65 ppm, (PMA–S–CH_2_–**C̲**(H_2_)–OH).

**Fig. 4:**
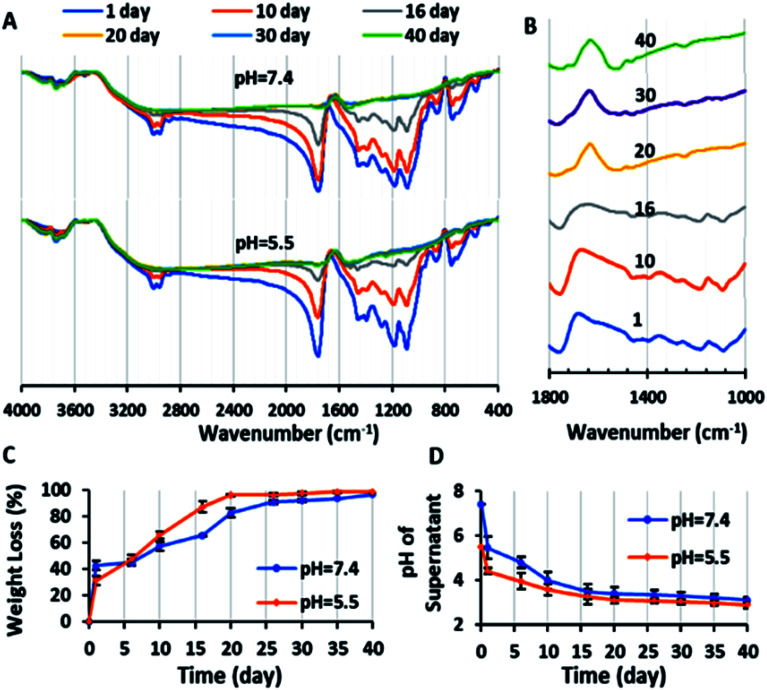
(A) FTIR spectra of copolymer during degradation test in PBS with pH values of 7.4 and 5.5 after 1, 10, 16, 20, 30 and 40 days. (B) FTIR spectra of copolymer in the wavenumber range of 1000–1800 cm^−1^ during the degradation test in PBS with a pH value of 7.4 after 1, 10, 16, 20, 30 and 40 days. (C) Weight loss percentage of the copolymer (with initial copolymer weight of 10 mg in 2 mL PBS with pH = 7.4 and 5.5) during the degradation test *versus* time: 1, 6, 10, 16, 20, 26, 30, 35 and 40 days. (D) Plot of variation in pH (pH of supernatant of degradation suspension after centrifuging) *versus* time with initial PBS pH = 7.4 and 5.5 (*n* = 2).

In Fig. 4-S,† in the ^1^HNMR spectrum of P2 (CA-*g*-PMA–OH), the polymaleate peaks are observed at: *δ* = 2.73 ppm, 1H, (–C**H̲**(CO–O–CA)–CH(COOH)–); *δ* = 2.95, 2.97 ppm, 2H, end (C**H̲**_2_(CO–O–CA)–CH(COOH)–) group of PMA; *δ* = 3.381 ppm, 1H, (–CH(CO–O–CA)–C**H̲**(COOH)–); *δ* = 3.617 ppm, 1H, end (CH_2_(CO–O–CA)–C**H̲**(COOH)) group of PMA. Also, the citric acid signal was seen in: *δ* = 2.77–2.82 ppm, 2H, (HO–(CH_2_)_2_–S–PMA–O–C (C**H̲**_2_–COOH)_2_ (COOH)). The peaks at *δ* = 2.08 ppm, 2H, (CA-*g*-PMA–S–C**H̲**_2_–CH_2_–OH); *δ* = 4–4.1 ppm, 2H, (CA-*g*-PMA–S–CH_2_–C**H̲**_2_–OH); *δ* = 5–5.5 ppm, 1H, (CA-*g*-PMA–S–CH_2_–CH_2_–O**H̲**) are related to the mercapto ethanol end group.

As can be seen in Fig. 4-S,† in the ^13^CNMR spectrum of P2, the peaks of PMA appear at: *δ* = 28.35 ppm, (–**C̲**H(CO–O–CA)–CH(COOH)–); *δ* = 44.55 ppm, (–CH(CO–O–CA)–**C̲**H(COOH)–); *δ* = 171.35 ppm, (–CH(**C̲**O–O–CA)–CH(COOH)–). The citric acid signals are located at: *δ* = 41.24 ppm, (HO–(CH_2_)_2_–S–PMA–O–C(**C̲**H_2_–COOH)_2_ (COOH)); *δ* = 71 ppm, (HO–(CH_2_)_2_–S–PMA–O–**C̲**(CH_2_–COOH)_2_ (COOH)); *δ* = 177.26 ppm, (HO–(CH_2_)_2_–S–PMA–O–C(CH_2_–**C̲**OOH)_2_ (COOH)). The signals of mercapto ethanol are present at: *δ* = 29.78 ppm, (PMA–S–**C̲**H_2_–C(H_2_)–OH); *δ* = 67.4 ppm, (PMA–S–CH_2_–**C̲**(H_2_)–OH).

According to the ^1^HNMR spectrum of P (CA-*g*-PMA-*co*-PLGA) in Fig. 3A, the signals of the lactide monomer of the PLGA tail are present at: *δ* = 1.27–1.34 ppm, 3H, (end CH_3_ group), *δ* = 1.47 ppm, 6H, (CH_3_); *δ* = 5–5.3 ppm, 2H, (CH); *δ* = 4–4.15 ppm, 2H, (end –C**H̲**(–CH_3_)–OH group); *δ* = 4.7 ppm, 1H, (end –CH(–CH_3_)–O**H̲** group). The glycolide peaks are observed at: *δ* = 4.96 ppm, 1H, (end –CH_2_–OH group); *δ* = 4.8–4.91 ppm, 4H, (CH_2_); *δ* = 4.28–4.48 ppm, 2H, (end –C**H̲**_2_–OH group). Also, the peaks of the maleate section appear at: *δ* = 2.81–2.98 ppm, 1H, (–C**H̲**(–(C=O)–O–CA)–CH(COOH)–); *δ* = 3.35–3.4 ppm, 1H, (–CH(–(C=O)–O–CA)–C**H̲**(–COOH)–); *δ* = 3.6 ppm, 1H, (–S–C**H̲**(–(C=O)–O–CA)–CH(–COOH)–); *δ* = 3.76–3.8 ppm, 1H, (–S–CH(–(C=O)–O–CA)–C**H̲**(–COOH)–). The citric acid signal is located at: *δ* = 2.61–2.69 ppm, 4H, (CH_2_). The peals of the mercapto section signatures are located at: *δ* = 2.53–2.57 ppm, 2H, (CA-*g*-PMA–S–C**H̲**_2_–CH_2_–O–PLGA); *δ* = 4.19–4.25 ppm, 2H, (CA-*g*-PMA–S–CH_2_–C**H̲**_2_–O–PLGA).

In the ^13^CNMR spectrum of P (Fig. 3B), the peaks related to the lactide monomer of the PLGA section are located at: *δ* = 16.47–16.55 ppm, (CH_3_); *δ* = 68.71–69.18 ppm, (CH); *δ* = 168.96–169.21 ppm, (–CH(CH_3_)–**C̲**(=O)–O–CH(CH_3_)–). The glycolide signatures appear at: *δ* = 60.69 ppm, (CH_2_); *δ* = 166.56–166.68 ppm, (–CH_2_–**C̲**(=O)–O–CH_2_–). Also, the maleate block signals are present at: *δ* = 20.32 ppm, (CH); *δ* = 172.07 ppm, (–CH(–**C̲**(=O)–O–CA)–CH(–C(=O)–OH)–); *δ* = 174.01 ppm, (–CH(–C(=O)–O–CA)–CH(–**C̲**(=O)–OH)–). The citric acid peaks are located at: *δ* = 47.25–47.34 ppm, (CH_2_); *δ* = 73.81 ppm, (MA–O–**C̲**–(COOH)–(CH_2_–COOH)_2_). The mercapto group signals are observed at: *δ* = 17.10–17.18 ppm, (CA-*g*-PMA–S–**C̲**H_2_–CH_2_–O–PLGA); *δ* = 64.88 ppm, (CA-*g*-PMA–S–CH_2_–**C̲**H_2_–O–PLGA).

The molar mass (*M*_n_) of the copolymer was determined using the ^1^HNMR spectrum by integrating the signals pertaining to each monomer based on the following equation:^39^
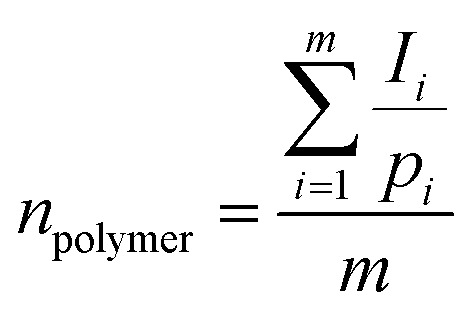
*M*_n_ = *n* × (monomer molecular mass)where *m* is the number of polymer signals, and *I*_*i*_ and *p*_*i*_ are the integration and number of protons related to the *i*^th^ polymer signal, respectively. The calculated molar mass of the copolymer (using ^1^HNMR spectra), and calculated and theoretical monomer ratio are listed in Table 2. The calculation details are shown in the ESI.†

**Table 2:** Calculated *M*_n_ value of the copolymer according to the ^1^HNMR spectra and calculated and theoretical monomer molar ratio of the copolymer

Parameter	*M*_n_ (g mol^−1^)	LA : GL %		LA : GL : CA-*g*-PMA %	
		Theoretical	Calculated	Theoretical	Calculated
Copolymer	2965.79	61.86 : 38.14	53.92 : 46.08	57.46 : 35.42 : 7.12	47.28 : 40.41 : 12.31

#### CHNS elemental analysis

The CHNS elemental analysis results are listed in Table 3. 4.249 mg copolymer was used for this analysis. The appearance of sulfur in the CHNS analysis of the copolymer confirmed the presence of sulfide linkages in the copolymer. A trace percentage (0.94%) of nitrogen appeared in the CHNS data, which is probably related to the DMF residue (as the solvent in the second step of the synthetic procedure). The CHNS plot is presented in Fig. 6-S in the ESI.†

**Table 3:** CHNS–O elemental analysis

CHNS–O	Elemental analysis (% w)				
	C	H	O	S	N
Copolymer	45.39	5.04	40.83	7.80	0.94

#### DSC test

According to the DSC curve of the copolymer (using second run data), as shown in Fig. 10-S,† the glass transition point (*T*_g_) of the copolymer was 50.2 °C. The melting point (*T*_m_) peak of the copolymer was not observed in the DSC curve; therefore, the copolymer had an amorphous structure and did not exhibit any crystallinity. Also, in similar reports on PLGA or PLGA-based copolymers, the *T*_m_ was not observed and the *T*_g_ value was determined to be about 40–60 °C, confirming this result.^40–44^

#### Copolymer degradation test

In the degradation process, the copolymer was expected to be hydrolyzed to its monomers in the PBS aqueous environment with time.^45^ These monomers including lactic, glycolic, maleic and citric acid have numerous carboxylic acid groups, are soluble in water and caused a decrease the pH of supernatant after the degradation of the copolymer. Thus, the variation in the pH of the PBS supernatant during the copolymer accumulated degradation was a satisfied factor in this study. According to Fig. 4D, the pH of the PBS supernatant decreased with time. The final pH of the supernatant was 2.89 and 3.1, with an initial PBS pH of 5.5 and 7.4, respectively. The sharp decrease in pH after the first day is related to the carboxylic acid groups of the copolymer, which were hydrolyzed in water as the copolymer dissolved. However, after 6 day, the neutral medium and the acidic medium resulted in the hydrolysis and degradation of the copolymer. After 16 days, the pH value reached a plateau with the final pH value of 3.1, which is much lower than the results in other reports on PLGA (pH ≈ 5.48–7.4, on day 16).^40,46^ This value confirmed the effect of the carboxylic acid groups of the CA-*g*-PMA sections on the approximately complete degradation of the copolymer on the 16^th^ day.

On the other hand, due to the degradation and fragmentation of the copolymer and presence of the degradation products in the medium, the weight of the copolymer decreased. This weight loss (WL%) is another factor that should be studied for degradation. Fig. 4C shows that the WL% increased with the degradation time. The weight loss in the first day is due to the dissolution of the copolymer in the PBS. After 10 days the WL% increased significantly and reached a plateau in 20 days. The final WL% was 96.75% and 98.5% for pH = 7.4 and 5.5, respectively.

Also, the chemical structure of the residue copolymer after different time intervals was studied *via* FTIR spectroscopy. As can be seen in Fig. 4A and B, the intensity of all the ester peaks decreased with the degradation time. After the 20^th^ day, the (C–O) stretching bands of the ester groups of the copolymer at 1087 and 1176 cm^−1^ disappeared because of the complete hydrolysis of the ester groups and the (C–O) stretching peaks of the carboxylic groups of the copolymer at 1390–1450 cm^−1^ shifted to the left (1461–1519 cm^−1^). Also, the (C=O) stretching single sharp peak at 1760 cm^−1^ decreased in magnitude and was duplicated (1745 and 1697 cm^−1^). This is may be due to the (C=O) stretching band of the aldehyde and ketone groups (as the probably degradation products), which are observed at lower frequencies (1700–1750 cm^−1^) in comparison to the ester bands (1755–1765 cm^−1^).^45,47^ In conclusion, the novel biodegradable copolymer in aqueous medium degraded faster than PLGA due to its carboxylic acid units, which may facilitate its hydrolysis *via* autocatalysis.^47^

### Micelle characterization, CMC, drug encapsulation and loading efficiency determination

The SEM image showed that the blank polymeric micelles (P) had a semi-spherical morphology with an average diameter of 51.9 nm (Fig. 11-S†), which is a desirable size for passing through the tumor leaky vasculature and passive targeting. The DLS-zeta data showed that the blank micelles have an average hydrodynamic diameter of 192.6 nm (PDI = 0.367) and zeta potential of around −29.7 mV (Fig. 8-S and 9-S†). The difference between the size of micelles from the DLS and SEM analysis can be explained by the copolymer being swollen with water molecules in the DLS analysis, which lead to a greater diameter than that observed by SEM.^48^ The acceptable PDI range is between 0.05–0.7, and thus the PDI of the micelles in this study of 0.367 is in this range, demonstrating their homogeneity.^49^ The acceptable size and PDI of the developed micelles increased their stability in the blood stream and improved their potential for intra-cellular uptake and deeper tissue penetration.^50^ A zeta potential of about ±30 can cause electrostatic stabilization, and subsequently increase the circulation time of nanoparticles in the blood; therefore, according to this claim, the zeta potential of the blank micelles (−29.7 mV) is suitable.^51^ This obtained zeta potential of P (−29.7 mV) is consistent with the results of other studies.^52^

**Fig. 5:**
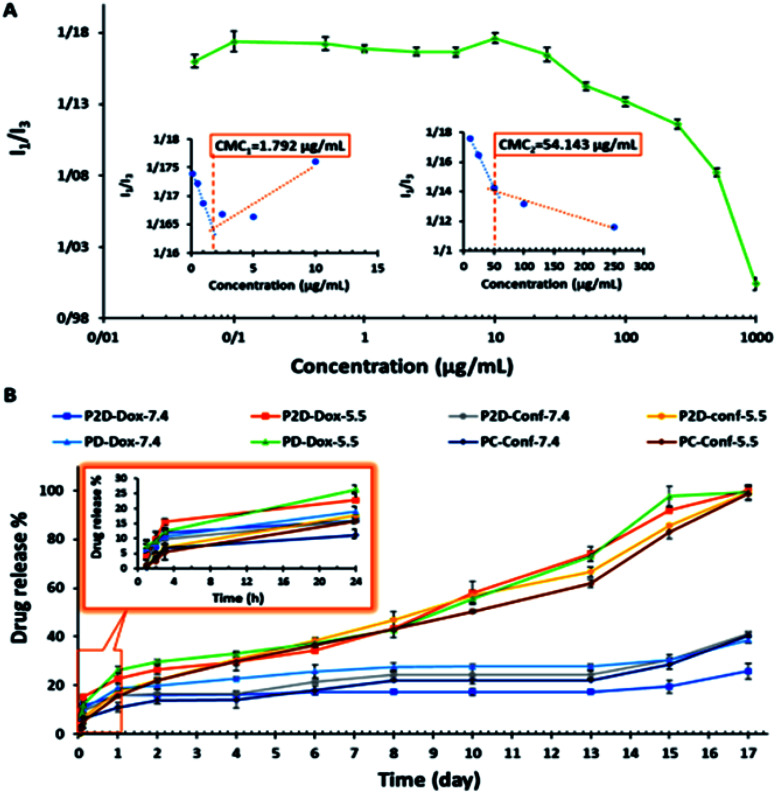
(A) Determination of CMC points of copolymer *via* spectrofluorometric method (pyrene probe), where copolymer concentration: 0.01, 0.05, 0.5, 1, 2.5, 5, 10, 25, 50, 100, 250, 500, and 1000 μg mL^−1^ in final solution, and pyrene concentration: 0.005 μg mL^−1^ in final solution. (B) Drug release percentage *versus* time (day) plot, time intervals: 1, 2, 4, 6, 8, 10, 13, 15 and 17 days, 2 mg nano-formulation in 2 mL sink solution with pH = 7.4 and 5.5.

The CMC of the blank micelles was determined by plotting copolymer concentration *versus I*_1_/*I*_3_ ratio (Fig. 5A). The decreasing in the *I*_1_/*I*_3_ ratio with an increase in the copolymer concentration showed that the pyrene was located in the hydrophobic core of the micelles. As can be observed in Fig. 5A, two minimum points are located after the maximum points, presenting CMC_1_ = 1.793 μg mL^−1^ and CMC_2_ = 54.143 μg mL^−1^ of the copolymer, respectively. This polydispersity in the CMC diagram (CMC_1_ and CMC_2_) occurred owing to the polydispersity of the copolymer and the statistical procedure of the self-assembly process.^53^ On the other hand, in many reports, this phenomenon was explained by the transformation of the micelle shape from spherical to cylindrical with an increase in copolymer concentration.^54^ According to these results, the acidic surface of the copolymer promoted micelle formation with a low CMC (CMC_1_ = 1.793 μg mL^−1^), confirming their dynamic stability in the blood stream.^55^

Also, functionalization of the polymeric micelles with carboxylic acid groups of citric and maleic acid enhanced the doxorubicin loading because of the increase in electrostatic interactions between the carboxylic acid groups of the micelles (p*K*_a_ = 5) and amine groups of Dox (p*K*_a_ = 8.3) at the physiological pH (7.4); however, it was also possible to load Dox into the micelle core. Conf was loaded into the micelle core almost completely due to its very hydrophobic property. The decrease in the zeta potential of the co-drug-loaded micelles (−6.57 mV) compared to the blank micelle (−29.7 mV) shows that Dox was loaded successfully on the surface of the micelles.

Dox, Conf and a combination of drugs (2D) were separately loaded into the micelles, where the weight ratio of drug to copolymer was 1 : 10. The drug encapsulation efficiency (DEE%) of the single-drug loaded nano-formulation (PD and PC) and co-drug loaded nano-formulation (P2D) is listed in Table 4. The drug encapsulation efficiency (DEE%) values show that the novel copolymeric micelles have very high capacity for drug loading. Thus, to confirm the drug loading in the micelles, the blank- and co-drug-loaded micelles (P and P2D, respectively) were analyzed *via* FTIR. According to Fig. 2-S-P2D,† the peak at 3445 cm^−1^ is assigned to the stretching of the (–OH) and (–NH) groups present in the copolymer and Dox, respectively. The characteristic peak of Dox (in plane bend of NH_2_ group) was observed at 1650 cm^−1^, which overlapped with the Conf (C–C) stretching of the aromatic ring group, confirming the loading of both drugs in the micelles.

**Table 4:** Drug encapsulation efficiency (DEE%) of the nanoformulations

Formulation	P2D	PD	PC
Dox	98.87	97.83	—
Conf	99.9	—	99.88

### Evaluation of *in vitro* release study

The *in vitro* release study of the single- (PD and PC) and co-drug-loaded (P2D) micelles was performed in a suitable sink solution for Conf release (99% PBS, 0.5% DMSO, and 0.5% Tween 20) at two different pH values, 7.4 and 5.5, at 37 °C.

The release results (Fig. 5B) indicate that initially (24 h), the formulation was not pH sensitive and did not exhibit a noticeable release. The small amount of release below 24 is may be due to the entrance of unloaded drug in to medium. However, after this period, the pH-sensitive property of the drug-loaded micelles and drug release percentage increased (Fig. 5B). At the physiological pH 7.4 (>micelles p*K*_a_ = 5), the hydrolyzed carboxylate groups of the copolymer had a negative charge, and the amine part of Dox (p*K*_a_ = 8.3) was protonated and had a positive charge. Therefore, the electrostatic interaction between the negatively charged copolymer and the positively charged Dox prevented the drug from being released in the medium. On the other hand, at pH 5.5, the carboxylic acid groups of the copolymer were protonated and neutralized, and thus Dox was released due to the disappearance of the copolymer–Dox ionic interaction. These results are consistent with release results of the mitoxantrone/doxorubicin loaded-pH sensitive *block*-polymer reported by Ramasamy *et al.*^56^ With regard to the big gap between the physiological and acidic pH release percentage of the drugs in all the formulations in this study, the new nano-formulations can be applied as a potent pH-sensitive drug carrier. Also, according to Fig. 5B, the abrupt drug release at both pH values after day 13^th^ occurred due to degradation of the copolymer, confirming the degradation results.

### Cell uptake study results

**Fig. 6:**
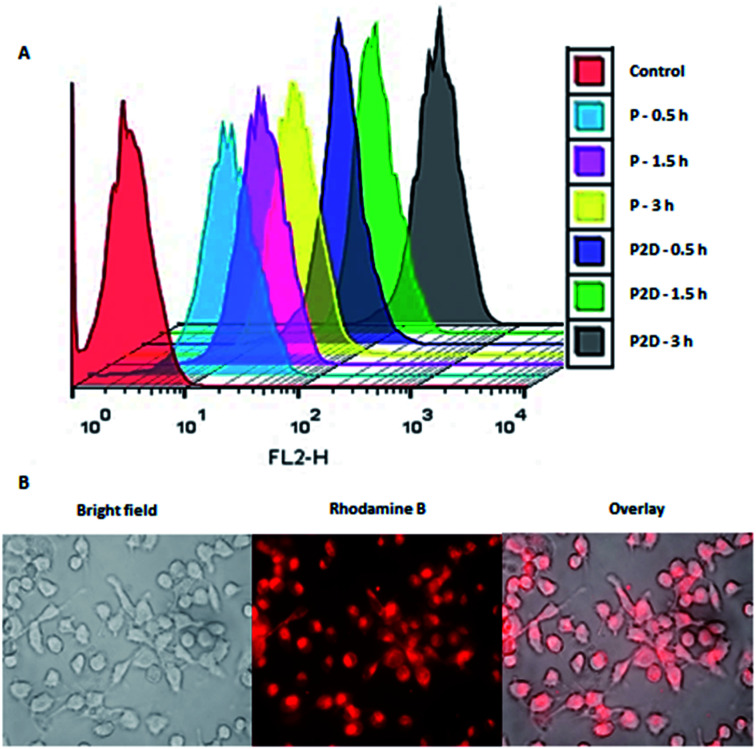
(A) Cellular uptake of RB-labeled P and P2D (10 and 1 μg mL^−1^, respectively) within 0.5, 1.5, and 3 h into MDA-MB-231 cells using flowcytometery. (B) Uptake images of RB-P2D micelles (1 μg mL^−1^) into MDA-MB-231 cells using a research fluorescence microscope.

The penetration of P and P2D into the MDA-MB-231 cell line was determined to evaluate the capability of the novel nanoformulation in increasing the accumulation of drugs in cells. As shown in Fig. 6A, the cellular uptake of P increased with time (33%, 60%, 81% for 0.5, 1.5 and 3 h, respectively). The remarkable uptake percentage of P within 3 h shows that the blank micelles had a favorable size and structure to penetrate the cells. According to Table 1-S,†P2D exhibited a good uptake percentage within all the time intervals (97%, 99.7%, 100% for 0.5, 1.5 and 3 h, respectively),showing the relatively fast uptake of this nano-formulation. The higher cellular uptake of P2D compares to P at 0.5 h is due to the decrease in the negative surface charge of P2D (−6.57 mV) compared to P (−29.7 mV), as determined by the zeta potential, because the lower negative charge has less electrostatic repulsion forces with the negative cell membrane, and therefore higher uptake into the cells.^57^ The cellular uptake of the P2D nano-formulation was confirmed using a research fluorescence microscope (Fig. 6B), which showed that the RB-P2D micelles were taken into the cells.

### Results of uptake of blank micelles and co-drug loaded micelles into MDA-MB-231 spheroids

The penetration and uptake of RB-P and RB-P2D into the MDA-MB-231 spheroids were quantified by flow cytometry and Hoechst 33342 dye, which stained the nucleus of the cells. This method was used by Tchoryk *et al.* to study the uptake and penetration of nanoparticles and Dox into HCT116 spheroids, and according to their data, the FACS flow cytometry categorized the cells into three groups based on the intensity of the Hoechst fluorescence in the cells, including unstained cells (core of spheroid), moderately stained cells (middle of spheroid) and brightly stained cells (rim of spheroid). These sections were selected using the FlowJo Software (v. 10).

**Fig. 7:**
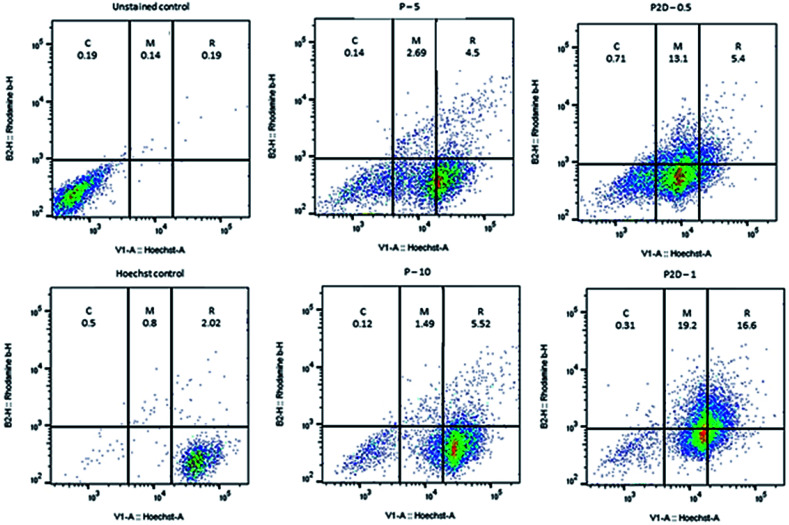
Plots of uptake amount of P (5 and 10 μg mL^−1^) and P2D (0.5 and 1 μg mL^−1^) into MDA-MB-231 spheroids by flow cytometry method, where the blank- and Hoechst-stained spheroids were used as the unstained and stained control, respectively. The uptake percentage was labeled on every section (C, M and R represent the core, middle and rim of the spheroid, respectively).

According to Fig. 7, 1.47%, 0.6% and 94.7% of the cell population were in the core, middle and rim of the Hoechst-stained spheroid (as the stained control), respectively. Also, the uptake and penetration amount of RB-P and RB-P2D into cells at different concentrations can be quantified based on Fig. 7. As shown in this figure, P (5 μg mL^−1^) penetrated and was taken up into the core, middle and rim of the spheroid by 0.14%, 2.69% and 4.5%, respectively. These amounts reached 0.12%, 1.49% and 5.5% with a concentration of 10 μg mL^−1^ of P. Also, P2D penetration and uptake into the core, middle and rim of the spheroid was 0.71%, 13.1% and 5.4% (0.5 μg mL^−1^) to 0.31%, 19.2% and 16.6% (1 μg mL^−1^), respectively. Upon comparing these results, it can be found that the penetration of both of P and P2D increased with an increase in their concentration, indicating that their uptake was concentration dependent. Also, the slight variation in uptake percentage (from lower concentration to higher concentration) may be due to the difference in the size of the spheroids, and subsequently in the counted cell number. On the other hand, it can be found that the P2D penetration and uptake percentage was higher compared to that of P at the same dose of P in the formulation. This result can be explained by the difference in the zeta potential of P (−29.7 mV) and P2D (−6.57 mV), where the lower negative charge of the P2D micelles causes higher cellular uptake because of the less repulsion forces with the negative charge of the cell membrane.

The low uptake percentage of P and P2D in the core of the spheroids can be explained by the large diameter of the spheroids (about 1000 μm). To achieve a higher uptake percentage, more time and smaller sized micelles are required. Therefore, a time-dependent analysis with a decrease in the micelle dimeter is proposed for future studies.

### *In vitro* cell viability study results

#### Cytotoxicity of nano-formulation on MDA-MB-231 cell line

**Fig. 8:**
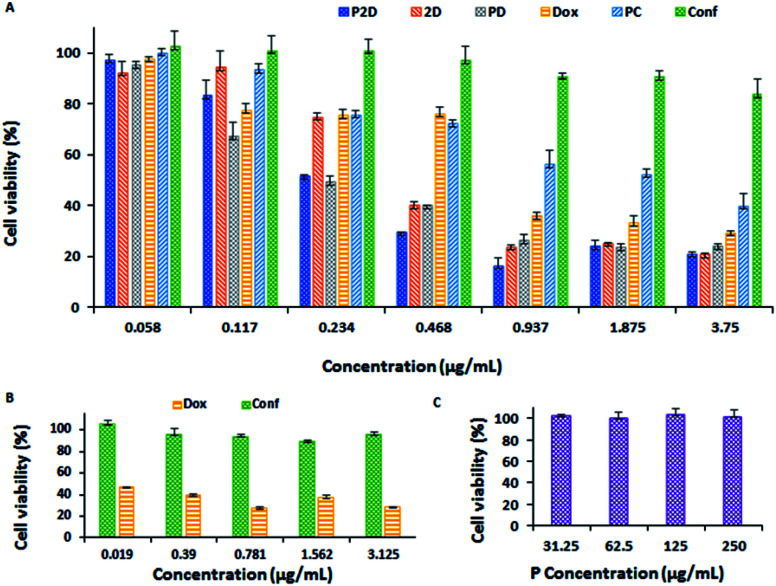
(A) Results of cytotoxicity of all the formulations (P2D, 2D, PD, Dox, PC and Conf concentration: 0.058, 0.117, 0.234, 0.468, 0.937, 1.875 and 3.75 μg mL^−1^) on the MDA-MB-231 cell line using the MTT method. (B) Results of Dox and Conf nephrotoxicity (on HEK-293 cell line) using the MTT method (Dox and Conf concentration: 0.019, 0.39, 0.781, 1.562, and 3.125 μg mL^−1^). (C) Blank micelles (P concentration: 31.25, 62.5, 125, and 250 μg mL^−1^) cytotoxicity on MDA-MB-231 cell line using the MTT method (all data was analyzed statistically using the Prism software, *n* = 3, and the differences between treatments was statistically significant, *p* < 0.0001).

The cytotoxicity of P, P2D, PD, PC, 2D, Dox and Conf on the MDA-MB-231 cell line was studied *via* the MTT method, and the results are shown in Fig. 8A. The cytotoxicity results for conferone at various doses are presented in Fig. 12-S.† The blank micelles (P) with different concentrations exhibited no significant cytotoxicity in 48 h (Fig. 8C), which shows that the micelles have no notable cytotoxicity on the MDA-MB-231 cell line. According to Fig. 8A, all the nano-formulations (P2D, PD, and PC) had more noticeable cytotoxicity than their related free drug form (2D, Dox, and Conf). Also, the combination forms (P2D and 2D) were more efficient than the related single forms (PD, PC, Dox and Conf). The IC_50_ values of all cases (P2D, PD, PC, 2D, Dox and Conf) were calculated using the Prism software, and shown in Table 5 and Fig. 13-S.† As can be seen in Table 5, the combination form (P2D) had an IC_50_ value of 0.198 μg mL^−1^ and this formulation was more effective with a lower Dox dosage in P2D (IC_50_ = 0.198 μg mL^−1^, containing 0.099 μg mL^−1^Dox and 0.099 μg mL^−1^Conf) in comparison with free Dox (IC_50_ = 0.569 μg mL^−1^), 2D (IC_50_ = 0.315 μg mL^−1^, containing 0.158 μg mL^−1^Dox and 0.158 μg mL^−1^Conf) and PD (IC_50_ = 0.157 μg mL^−1^). This means that in the combination nano-formulation, the highest antitumor efficacy was obtained with the lowest Dox concentration among the treatment groups. Also, with a decrease in the concentration of Dox, the toxic side effects were diminished. The IC_50_ of Conf was 33.683 μg mL^−1^, which is higher than that of PC (IC_50_ = 1.802 μg mL^−1^), owing to its water-insolubility, and therefore lower cellular uptake of Conf. All the result differences were statically significant (*P*_value_ < 0.0001).

**Table 5:** IC_50_ dosages of all the formulations calculated using the Prism v.8 software, *P*_value_ < 0.0001

Formulation	P2D	2D	PD	Dox	PC	Conf
IC_50_ (μg mL^−1^)	0.198	0.315	0.157	0.569	1.802	33.683

Also, the combination index (CI) was calculated using the CompuSyn software (v. 1) (Fig. 14-S and Table 2-S†). Considering that the CI of P2D and 2D was about 0.5 and 0.367 (<1) in related IC_50_ dosage, respectively, it can be concluded that the combination of drugs had a synergic effect on the IC_50_ dosage. An increase in P-glycoprotein (P-gp) expression and resultant resistance to chemotherapy are detected in progressive breast cancer tumors, which are usually characterized by a lower accumulation of drugs compared to sensitive tumors. Conferone inhibits P-gp-mediated drug efflux in breast cancer cells, which causes chemosensitivity in the cells, and subsequently, drug accumulation. Although conferone has low toxicity, it promotes the accumulation of Dox in cells *via* the inhibition of P-gp. Consequently, the superior cytotoxicity of P2D is due to both the synergic effect of the drugs and the enhanced accumulation of doxorubicin induced by conferone, as reported by Iranshahi *et al.*^58^

#### Nephrotoxicity of doxorubicin and conferone

To compare the nephrotoxicity of Dox and Conf at the same dosage on the HEK-293 cell line as a side-effect factor in chemotherapy, the MTT method was used (Fig. 8B). As can be seen in Fig. 8B, Dox had higher cytotoxicity on the renal cells at all the considered dosages (0.019, 0.39, 0.781, 1.562, and 3.125 μg mL^−1^) compared to Conf. For example, at the lowest dosage of the drugs (0.019 μg mL^−1^), the HEK-293 cell viability after 48 h was 46.6% and 100% for Dox and Conf, respectively.

Considering all the results of the MTT test, the P2D formulation had more notable cytotoxicity on the MDA-MB-231 cell line at a much lower dosage than the other formulations, especially free Dox and PD, and Dox exhibited remarkable nephrotoxicity compared to Conf at the same dosage.

### Cell cycle arrest results

DNA duplication in the replication of cells has four stages, G_1_, S, G_2_ and M,^59^ and the cell cycle test can be used to evaluate this phenomenon; therefore, the impact of all the formulations on the proliferation of the MDA-MB-231 cells was studied using the cell cycle assay.

**Fig. 9:**
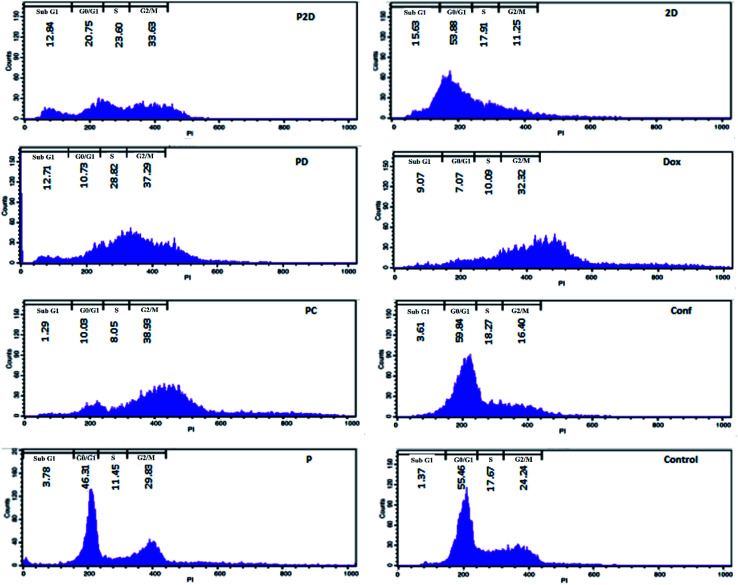
Cell cycle results for the different formulations (P2D, 2D, PD, Dox, PC and Conf, with a concentration of 0.198 μg mL^−1^) and blank micelles with a concentration of 1.98 μg mL^−1^ on the MDA-MB-231 cell line using flow cytometry. Untreated cells were considered as the control.

The results of the cell cycle analysis of all the formulations (P, P2D, PD, PC, 2D, Dox and Conf) on the MDA-MB-231 cells are shown in Fig. 9 and (Table 3-S†). The blank micelles (P) did not change the cell cycle pattern of the MDA-MB-231 cells in comparison with the control cells, confirming their safety.

The cells treated with the P2D and PD nano-formulations exhibited S (P2D: 1.33 and PD: 1.35-fold), G_2_/M (P2D: 1.39 and PD: 1.54-fold) and sub-G_0_ (P2D:9.37 and PD:9.28-fold) arrest, which presented a vigorous inhibitory outcome on DNA duplication and matched that of apoptotic cells.

The highest G_2_/M (1.6-fold) arrest was observed in PC. Conf with slight G_0_/G_1_ (1.08-fold) and S (1.03-fold) arrest, which may be due to the inadequate Conf cell uptake as a result of its high hydrophobicity, insufficient concentration of Conf (sub IC_50_ dose was used) and time-dependent function of Conf, which is consistent with results reported by Cheraghi *et al.*^19^Dox caused an increase in the G_2_/M (1.33-fold) and sub-G_0_ (6.62-fold) phases, but sub-G_0_ was dominant, in agreement with that reported by Sabzichi *et al.*^16^

As could be understood from the above results, the nano-formulations (P2D, PD and PC) acted more efficiently than their related free drug forms (2D, Dox and Conf, respectively) in disturbing the cell cycle, consequently leading to cell apoptosis.^60,61^

### Cell apoptosis results

**Fig. 10:**
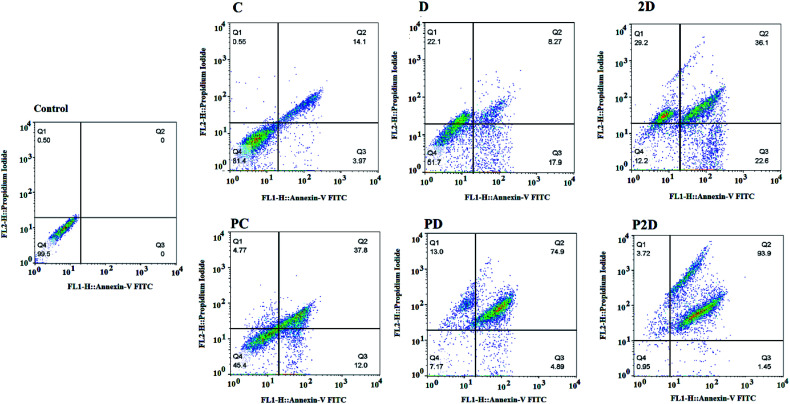
Apoptosis analysis results of the treated MDA-MB-231 cell line with the different formulations (Conf, PC, Dox, PD, 2D, and P2D, with a concentration of 0.198 μg mL^−1^) using flow cytometry. Untreated cells were considered as the control.

The capability of the apoptotic effect of all the formulations was investigated using the Annexin-V/propidium iodide (PI) double staining flow cytometry test, where Annexin-V was used as a fluorescent probe for apoptotic cells and PI stained the nucleus of the late apoptosis and necrotic cells.^62^ The results of the apoptosis test are presented in Fig. 10 and Table 4-S.† According to Table 4-S,† the cells treated with Dox and 2D showed more necrosis (22.1% and 29.2%, respectively) compared to that with PD and P2D (13% and 3.72%, respectively). Also, the percentage of apoptotic cells in all the nano-formulations (P2D, PD and PC) increased significantly compared to the necrotic cells. These results show that the nano-formulations programmed cell death through apoptosis in comparison to the free drugs. Among the nano-formulations, P2D exhibited the highest percentage of apoptosis (95.35%), demonstrating its powerful apoptotic programmed cell death action compared to the other treatment groups. The higher apoptosis ratios for the nano-formulations compared to the related free drugs was induced by their higher cellular uptake, and therefore higher cytotoxicity *via* apoptosis. Also, the highest percentage of apoptosis in the cells treated with P2D verified the synergistic effect of the drugs in this nano-formulation (CI = 0.5 obtained from combination index results) and Conf role on inhibition of Dox efflux. Thus, these results confirm the cell cycle outcomes, where the nano-formulations, especially P2D, induced apoptosis by sub-G_0_ and G_2_/M arrest, which stopped cell cycle progression.

### Realtime-PCR results

To investigate the mechanism of apoptosis in the treated MDA-MB-231 cells, the role of the caspase-dependent programmed cell death pathways was evaluated. The caspases (cysteine protease enzymes) are necessary for apoptosis, and some of them are initiator caspases (such as caspase-8 and 9) and others are effector caspases (for example caspase-3 and 7).^60^

**Fig. 11:**
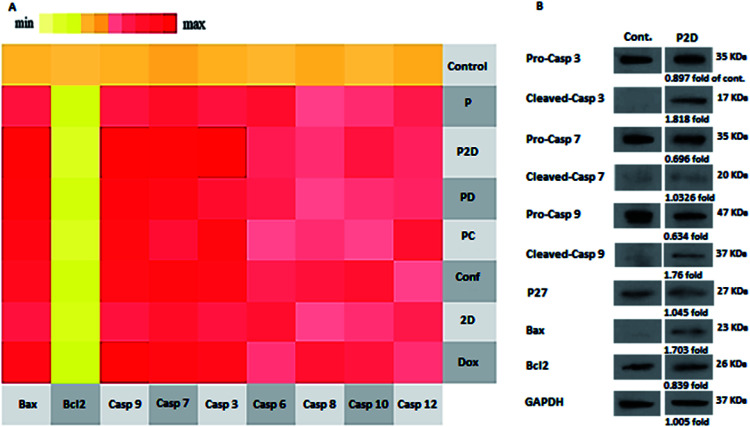
(A) qPCR expression of the apoptosis panel. The heat map indicates the range of expression of the apoptosis pathway-mediated genes in the cells treated with the different formulations (P2D, PD, PC, 2D, Dox and Conf, with a concentration of 0.1 μg mL^−1^) and blank micelles (P) with a concentration of 1 μg mL^−1^. Untreated cells were considered as the control. Bcl-2 indicates down-regulation in almost in all samples; however, the expression of Bax, Casp3, Casp7, and Casp9 was up-regulated, suggesting the activation of the intrinsic apoptosis pathway. (B) Western blotting results of P2D (0.1 μg mL^−1^), using Bax, Bcl-2, pro-Casp-3, cleaved-Casp-3, pro-Casp-7, cleaved-Casp-7, pro-Casp-9, Cleaved-Casp-9 and P27 genes and GAPDH as the internal control.

Also, Bax and Bcl-2 are the pro-apoptotic and anti-apoptotic proteins, respectively, which control the mitochondrial release of cytochrome-c.^63^ The heat map of the qPCR fold changes is presented in Fig. 11A. The blank micelles (P) did not show a significant apoptotic effect, which confirmed their non-toxicity property. Bcl-2 was down-regulated in all the formulations, which if accompanied by the up-regulation of Bax, lead to the release of cytochrome-c by the mitochondria. Bax up-regulation occurred in the following order: P2D > PD > Dox > PC > Conf > 2D. The mentioned Bcl-2/Bax change that caused caspase-9 activation was up-regulated as follows: P2D > Dox > PD > PC > Conf > 2D. Also, the activation of caspase-9 led to caspase-3 and 7 activation, which induced apoptosis in the cells. Also, in Dox, Conf and PC, caspase-8 and 12 were up-regulated slightly, but the up-regulation of caspase-9 in PC and Dox and caspase-7 in Conf was dominant. The qPCR results for the apoptosis pathway genes indicated that the expression pattern favors the intrinsic apoptosis pathway through the Bax/Bcl-2-caspase-9-caspase-3/caspase-7 axis. This axis is a sign of an intrinsic (mitochondria mediated) pathway, especially with regard to the suppression of caspase-8 (indicates an extrinsic pathway). Among the formulations, P2D caused the strongest promotion of caspase-dependent apoptosis, which confirms the cell-apoptosis results in the previous section.

### Western blot results

The real-time PCR results showed that the P2D had the largest effect on the caspase-dependent apoptosis in the MDA-MB-231 cell line at the gene level. Thus, to confirm the RT-q-PCR results, the influence of P2D on Bax, Bcl-2, pro-caspase-3, cleaved-caspase-3, pro-caspase-7, cleaved-caspase-7, pro-caspase-9, and cleaved-caspase-9, was assessed at the protein level by western blotting. Also, the effect of P2D on p27 (cyclin-dependent kinase inhibitor or KIP_1_) was evaluated because P27 acts as a cell cycle inhibitor and the concurrent up-regulation of P27 and Bax can cause apoptosis.^64^ The western blotting results are shown in Fig. 11B. According to the results (Table 5-S†), the expression of Bcl-2 (0.839-fold), pro-caspase-3 (0.89-fold), pro-caspase-7 (0.696-fold) and pro-caspase-9 (0.634-fold) was reduced in comparison to that of the control group. On the other hand, the expression of Bax (1.7-fold), cleaved-caspase-3 (1.82-fold), cleaved-caspase-7 (1.033-fold), cleaved-caspase-9 (1.76-fold) and P27 (1.045-fold) increased in comparison to that of the control group. The up-regulation of P27 showed that the cell cycle was stopped or disturbed and induced apoptosis by increasing Bax expression. Bax up-regulation with a decrease in Bcl-2 expression caused the up-regulation of cleaved-casp9, cleaved-casp3 and cleaved-casp7, which led to an increase in the cleavage of the death substrate and DNA fragmentation.^65^ According to the abovementioned pathway, the up-regulation of the cleaved-casp9, cleaved-casp3 and cleaved-casp7 proteins in the treated MDA-MB-231 cells with P2D was verified by apoptosis of the cells. Therefore, together with the RT-qPCR results, western blotting showed that P2D promoted caspase-dependent apoptosis through the Bax/Bcl-2-cleaved-caspspase-9/cleaved-caspase-3/cleaved-caspase-7 axis. This outcome is similar to that reported by Wei *et al.*, where the combination of magnoflorine with doxorubicin led to apoptosis in MDA-MB-231 breast cancer cells *via* the Bax/Bcl-2/cleaved-caspase-9/cleaved-caspase-3 axis.^66^

## Conclusions

In this study, novel pH-sensitive biodegradable micelles were engineering from citric acid-grafted polymaleate-*block*-PLGA with a very low CMC, homogenous spherical morphology, and high biodegradation rate. The engineered micelles were used for the first time for the combined delivery of doxorubicin (Dox) and conferone (Conf). These drug-loaded micelles showed sustained and high pH-sensitive release *in vitro*. The co-drug-loaded micelles (P2D) had higher and fast cellular uptake in comparison to the blank micelles (P) in both MDA-MB-231 cells (2D) and 3D cell culture models (spheroids). All the drug-loaded nano-formulations, especially P2D, had higher cytotoxicity compared to the free drugs with an equal Dox dosage on the MDA-MB-231 breast cancer cell line according to the MTT assay, but, P did not show any cytotoxicity. The combined form of Conf and Dox in P2D and 2D showed a high synergistic effect. Additionally, P2D resulted in cell cycle arrest, causing cell cycle inhibition and sequence cell apoptosis, as proven by the Annexin-V apoptosis test. Correspondingly, P2D induced apoptosis *via* the caspase-dependent intrinsic axis, which was proven at the gene and protein level by RT-qPCR and western blotting, respectively. In conclusion, the novel engineered Dox–Conf-loaded micelles (P2D) may provide a promising anti-cancer delivery system with reduced side effects, which should be investigated *in vivo* in the future because of all the above mentioned abilities.

## Conflicts of interest

Authors have no conflict of interest.

## Supplementary Material

RA-010-D0RA03467C-s001
