# Supplementary material for: Novel pH-sensitive and biodegradable micelles for the combined delivery of doxorubicin and conferone to induce apoptosis in MDA-MB-231 breast cancer cell line
Source: RSC Adv. 2020 Aug 7;10(49):29228–46. doi: 10.1039/d0ra03467c (PMC9055950; doi:10.1039/d0ra03467c)
Supplement: RA-010-D0RA03467C-s001 [file RA-010-D0RA03467C-s001.pdf]

Electronic Supplementary Material (ESI) for RSC Advances.
This journal is © The Royal Society of Chemistry 2020

Electronic Supplementary Information (ESI) available: [details of any supplementary information available should be included here]. See DOI: 10.1039/x0xx00000x

Supplementary file of:

Novel pH-sensitive and biodegradable micelles for combination delivery of Doxorubicin and Conferone to induce apoptosis in MDA-MB-231 breast cancer cell line

Akram Rahmani [a], Hassan Zavvar Mousavi [a,b*], Roya Salehi [c,d*], Ahmad Bagheri [a]

[a.] *Department of Applied Chemistry, Faculty of Chemistry, Semnan University, Semnan, Iran.*
[b.] *Department of Chemistry, Faculty of Science, University of Guilan, Rasht, P.O. Box 41335-1914, Rasht, Iran, E-mail-1: hzmousavi@semnan.ac.ir and E-mail-2: hzmousavi@guilan.ac.ir.*
[c.] *Drug Applied Research Center, Tabriz University of Medical Sciences, Tabriz, Iran, Tabriz, Iran, E-mail: salehiro@tbzmed.ac.ir.*
[d.] *Department of Medical Nanotechnology, School of Advanced Medical Sciences, Tabriz University of Medical Sciences, Tabriz, Iran.*

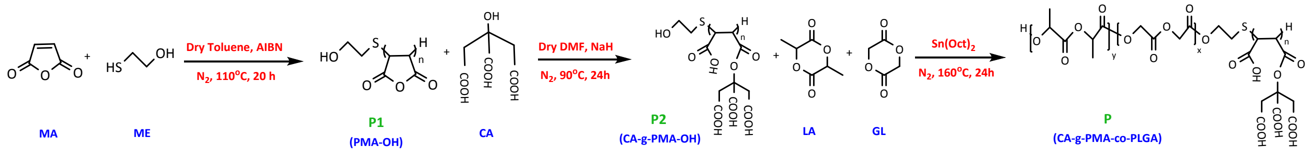

*Figure 1-S: synthesis root of copolymer, Step 1: Synthesis of hydroxy terminated poly maleic anhydride (**P1**: PMA-OH), Step 2: Functionalization of PMA-OH with citric acid (**P2**: CA-g-PMA-OH), Step 3: Post co-polymerization of CA-g-PMA-OH with Lactide and Glycolide (**P**: CA-g-PMA-co-PLGA).*

*[*]This figure is the same with Figure 1 in paper but in *.cdx file type.*

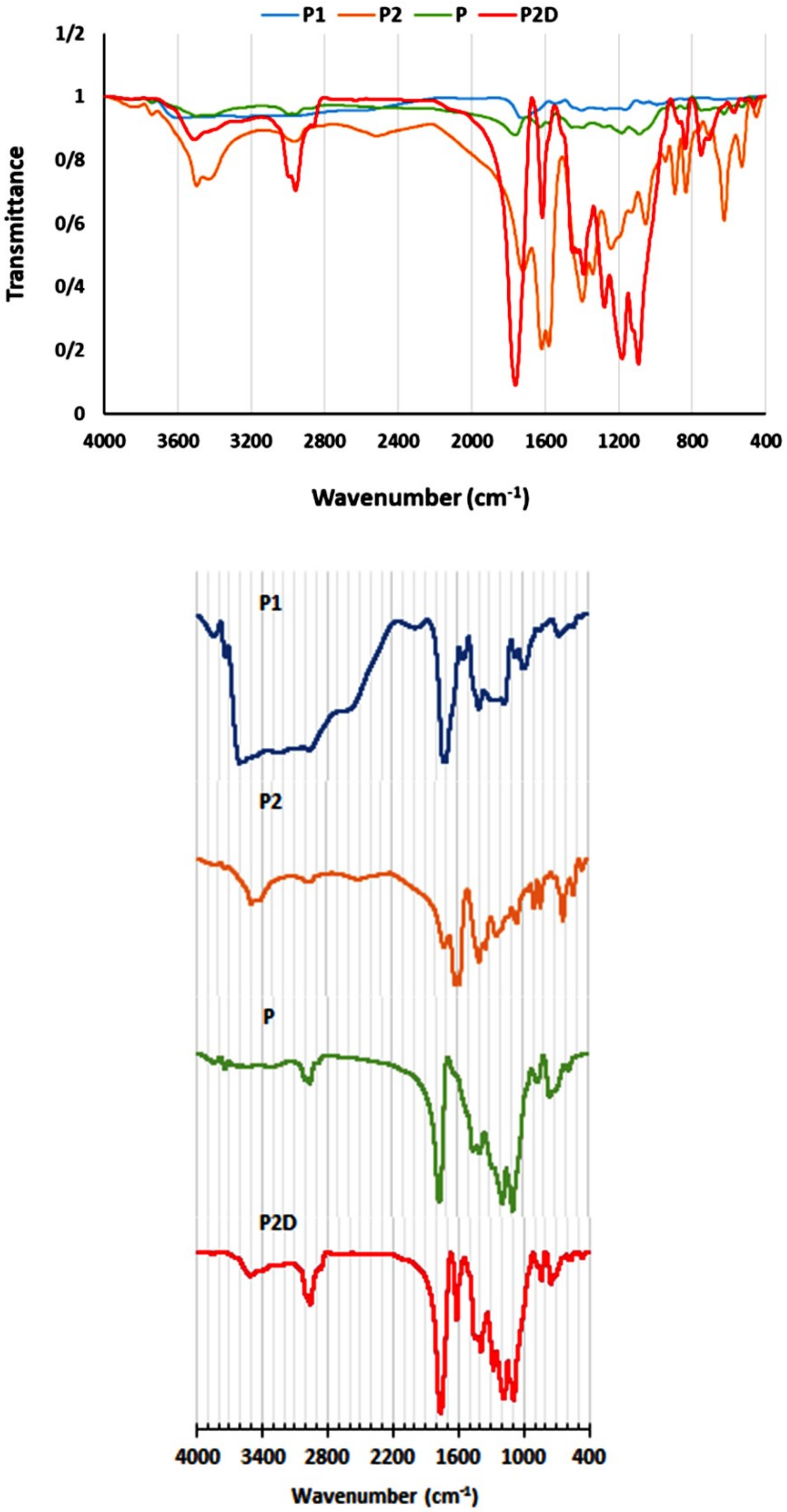

*Figure 2-S: FTIR Spectra of: **P1**: (PMA-OH), **P2**: (CA-g-PMA-OH), **P**: (CA-g-PMA-co-PLGA), **P2D**: (Co-drug loaded copolymeric micelles).*

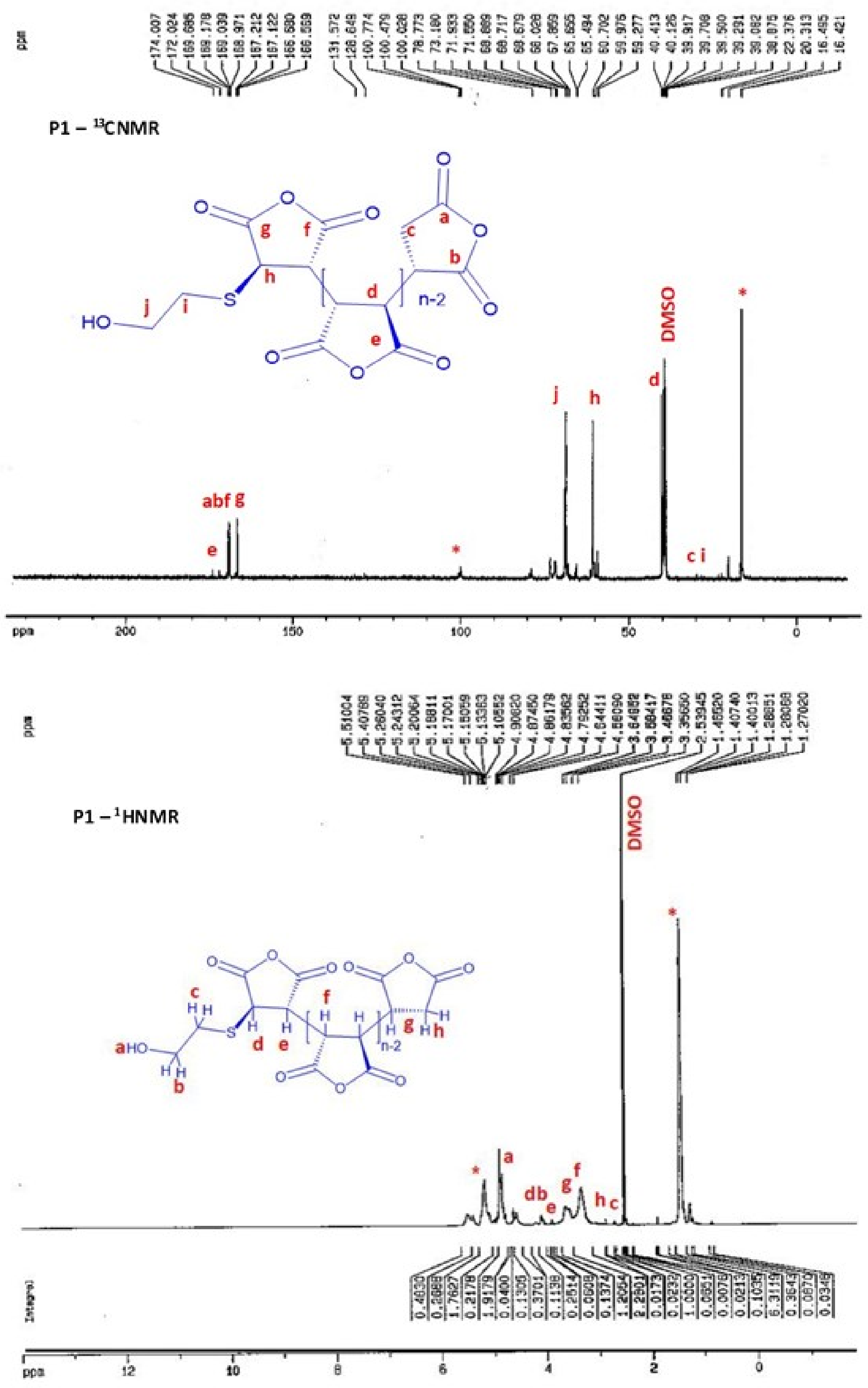

Figure 3-S: NMR spectra of ***P1****:* ***^{13}CNMR*** *spectra, that * are the peaks related to solvent residu****e and ^{1}HNMR*** *Spectra of* ***P1****, that * are the peaks related to free mercapto ethanole.*

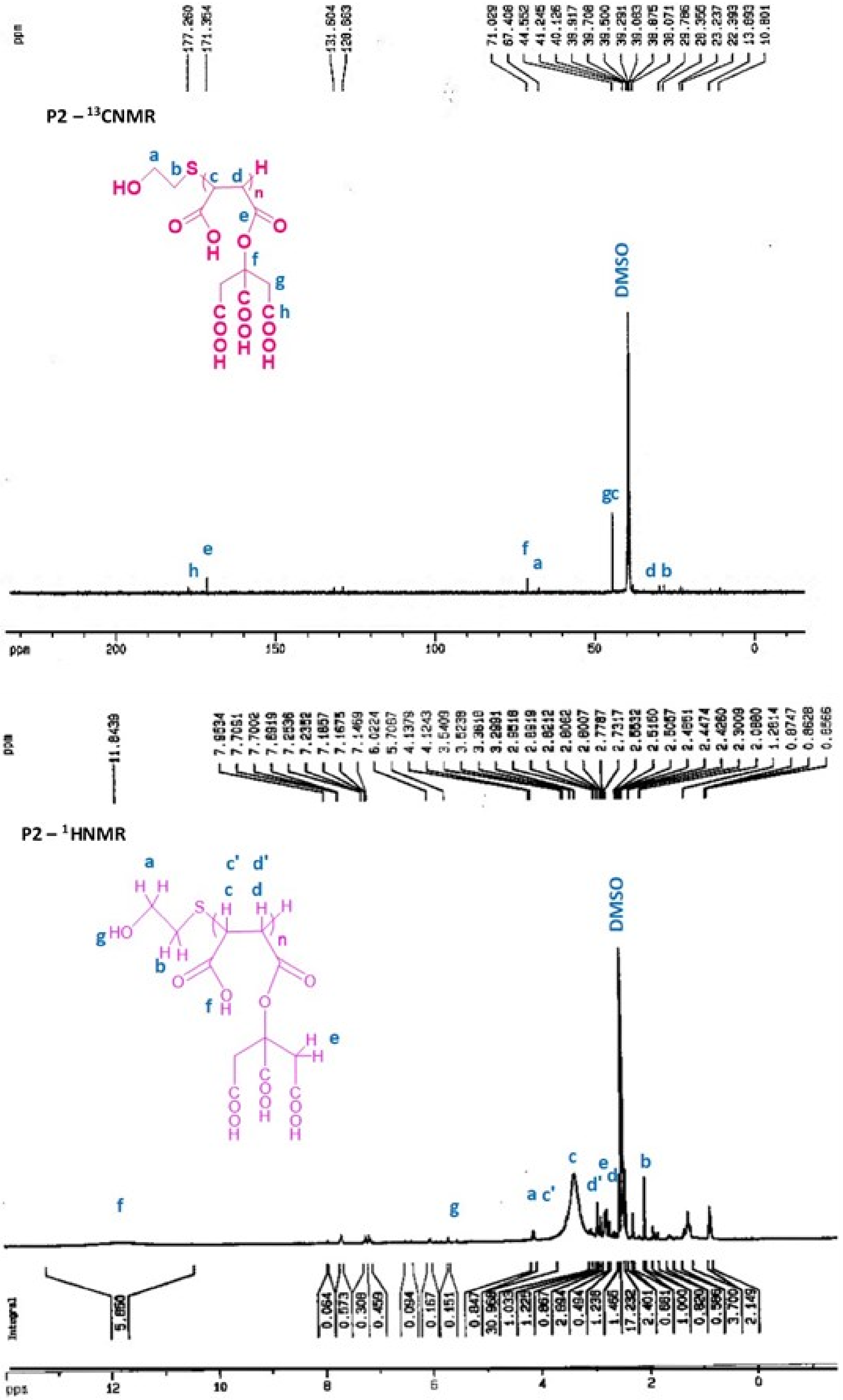

*Figure 4-S: NMR spectra of **P2**: [13]**CNMR** spectra of **P2**, **and** [1]**HNMR** Spectra of **P2**, that c' and d' are the peaks related to the signals pertained to maleate group that was banded to -S.*

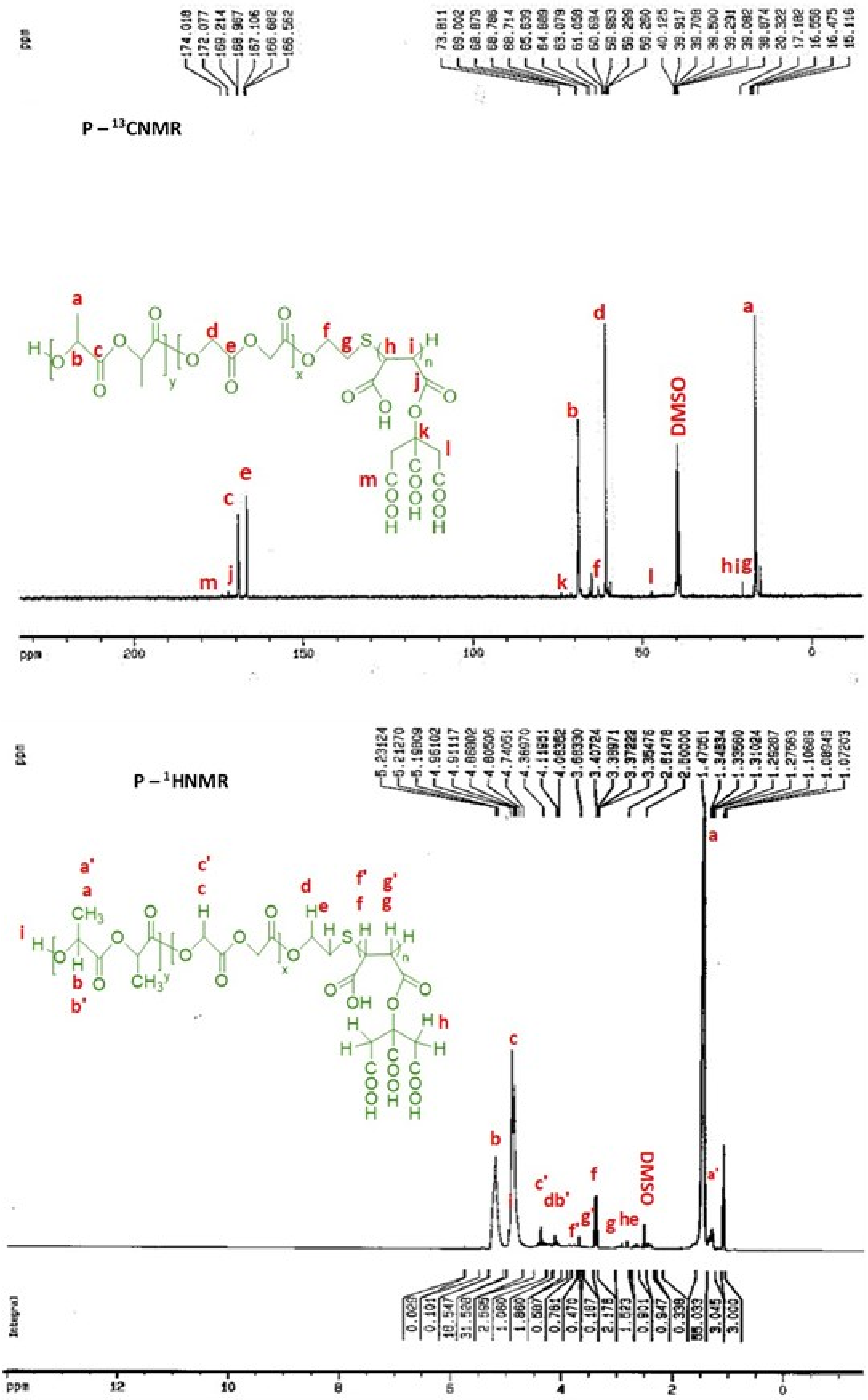

*Figure 5-S: NMR spectra of **P**: **^{13}CNMR** spectra of **P**, **and ^{1}HNMR** Spectra of **P**, that a', b' and c' are the peaks related to end groups, and f' and g'are the signals pertained to maleate group that was banded to -S.*

Calculation of molar mass of copolymer by ^{1}HNMR spectra

The molar mass (M_n) of copolymer was determined with ^{1}HNMR spectra, by integrating the signals pertaining to each monomer using following equation [Jay Wm. Wackerly, J. Chem. Educ]:

$$n_{polymer} = \frac{\sum_{i=1}^{m} \frac{I_i}{p_i}}{m}$$

$$M_n = n.\,(monomers\ molecular\ mass)$$

Where, *m* is the number of polymer signals that used, I_i and p_i are the integration and number of protons related to *i*th polymer signal.

$$n_{Lactide} = \frac{\frac{55.03}{6} + \frac{18.542}{2}}{2} = 9.22$$

$$n_{Glycolide} = \frac{\frac{31.528}{4}}{1} = 7.882$$

$$n_{MA} = \frac{\frac{2.176}{1}}{1} = 2.176$$

$$n_{CA} = \frac{\frac{0.901}{4}}{1} = 0.225$$

$$n_{ME} = \frac{\frac{1.0596}{2}}{1} = 0.53$$

$$n_{GL\ end\ group} = \frac{\frac{2.5951}{2}}{1} = 1.297$$

$$n_{LA\ end\ group} = \frac{\frac{1.86}{1}}{1} = 1.86$$

$$M_n = (9.22 \times 144.13) + (7.882 \times 116.07) + (2.176 \times 98.06) + (0.225 \times 210.14) + (0.53 \times \text{[illegible]} + (1.86 \times 144.13) + 1 = 2965.43\ g/mol$$

$$M_n = (9.22 \times 144.13) + (7.882 \times 116.07) + (2.176 \times 98.06) + (0.225 \times 210.14) + (0.53 \times \text{[illegible]} + 1 = 2670.717\ g/mol$$

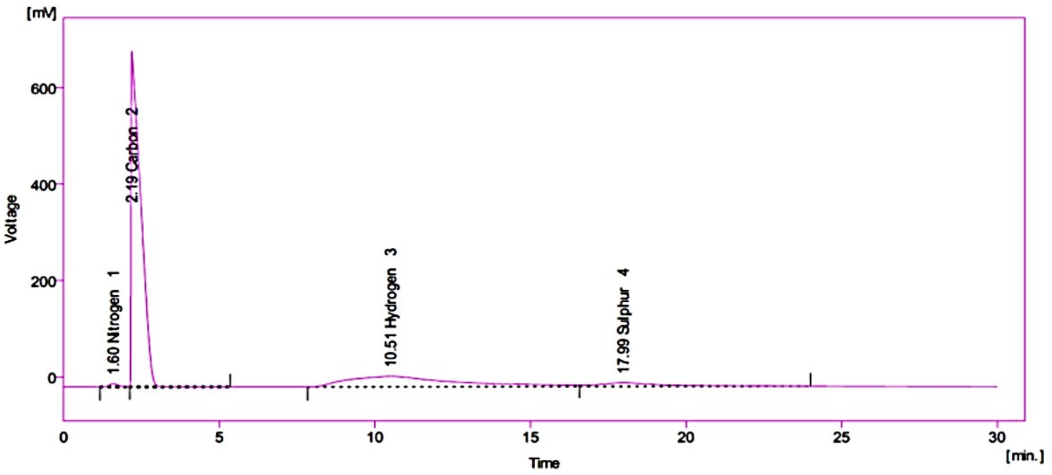

Figure 6-S: CHNS elemental analysis: plot of time (min) versus voltage (mV)

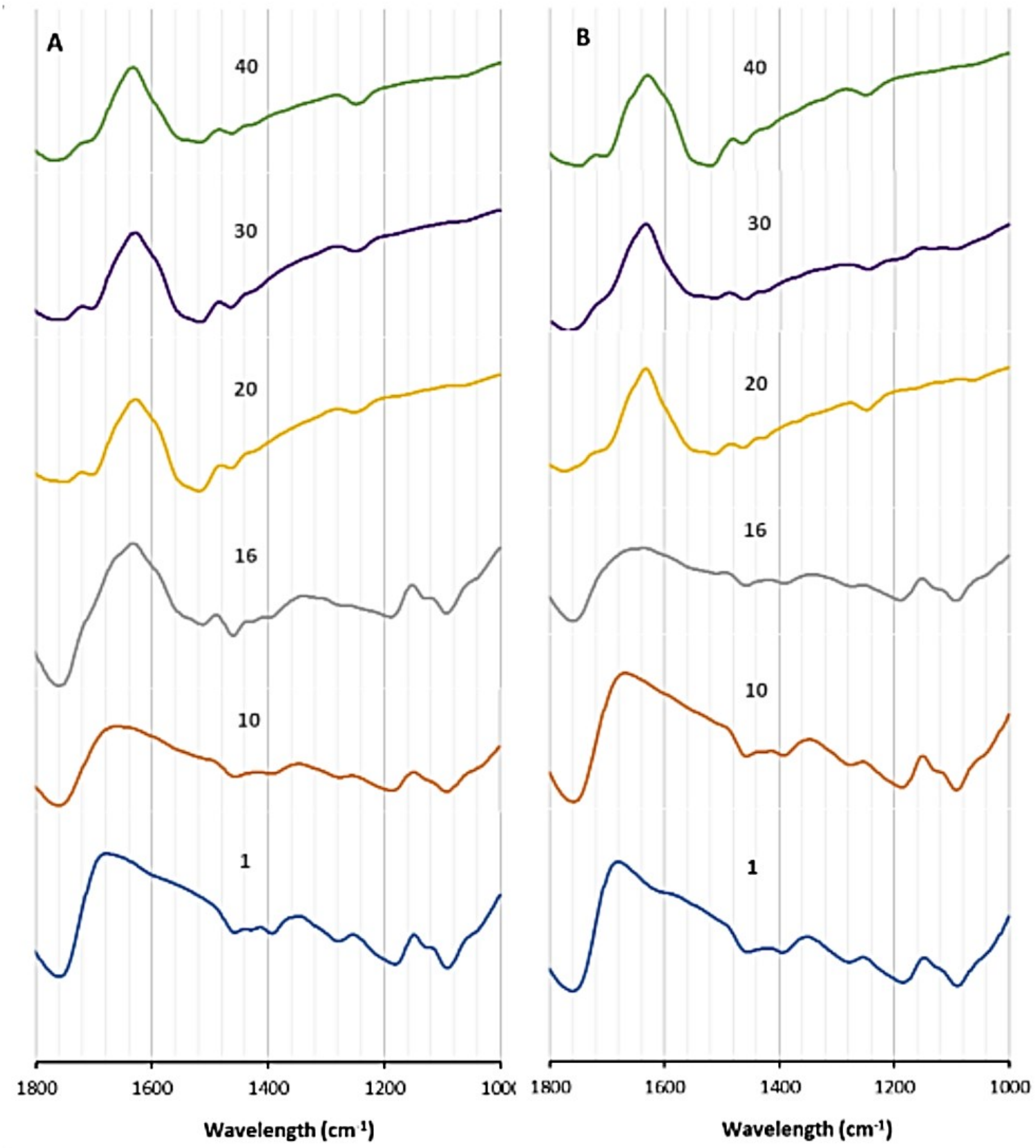

Figure 7-S: Copolymer FTIR spectra during degradation test after 1, 10, 16, 20, 30 and 40 day, wavenumber between 1000-1800 cm^{-1}, in PBS with pH value of A) pH = 5.5; B) pH = 7.4.

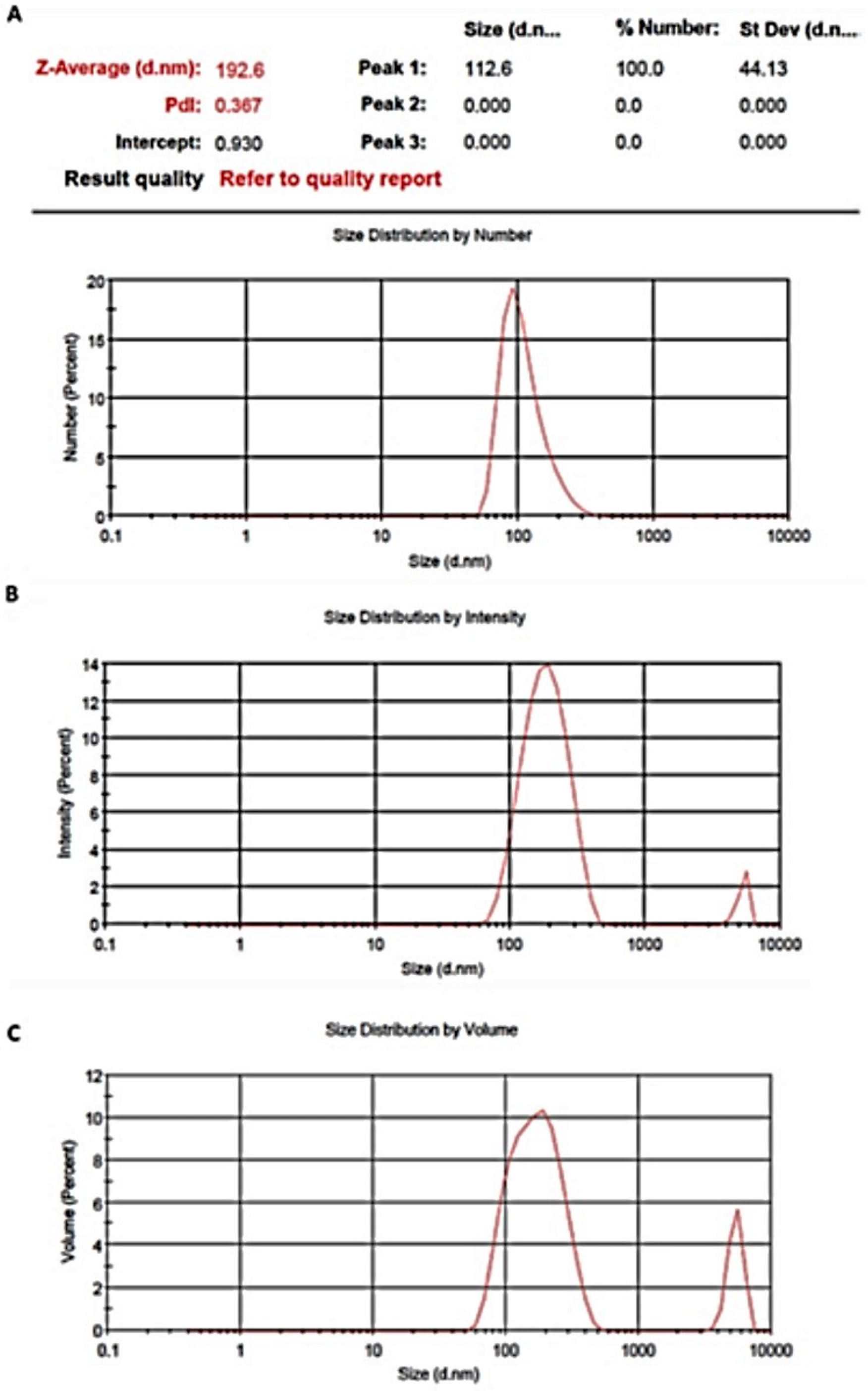

Figure 8-S: DLS results of P, A) number percent versus size (nm); B) Intensity percent versus size (nm); C) Volume percent versus size (nm).

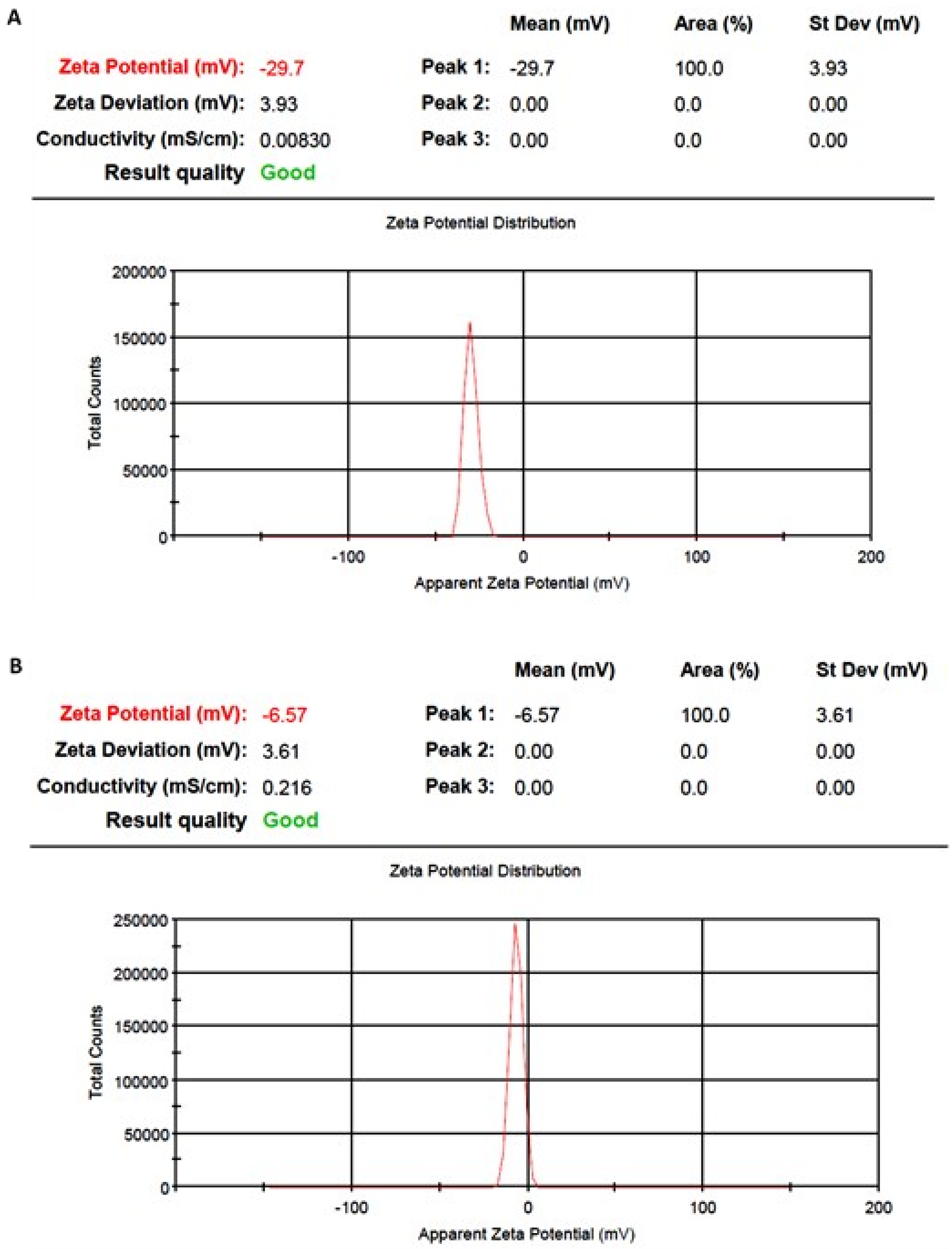

*Figure 9-S: Results of zeta potential (mV) measurments for: A) Blank micelles (**P**) ; B) Co-drug loaded micelles (**P2D**)*

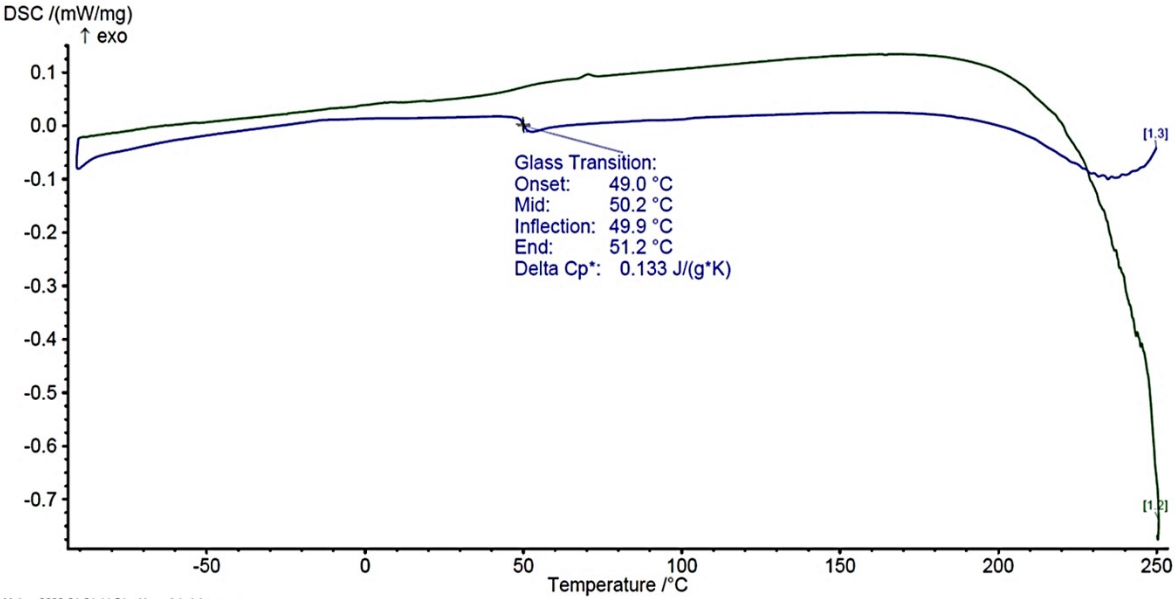

Figure 10-S: DSC plot of copolymer, plot of temperature vs. DSC.

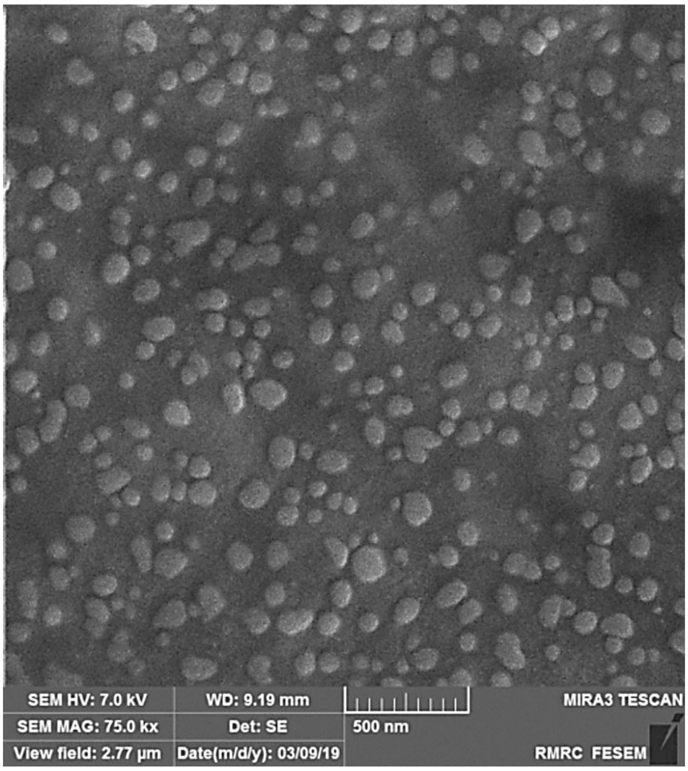

*Figure 11-S: SEM image of blank micelles (**P**)*

Table 1-S: *Percentage of cellular uptake of RB-P and RB-P2D micelles in 0.5, 1.5, and 3 h into MDA-MB-231 cell line using flowcytometery.*

**Cellular uptake %**	**Time (h)**		
	**0.5**	**1.5**	**3**
**P**	33.2	60.5	81.5
**P2D**	97.0	99.7	100

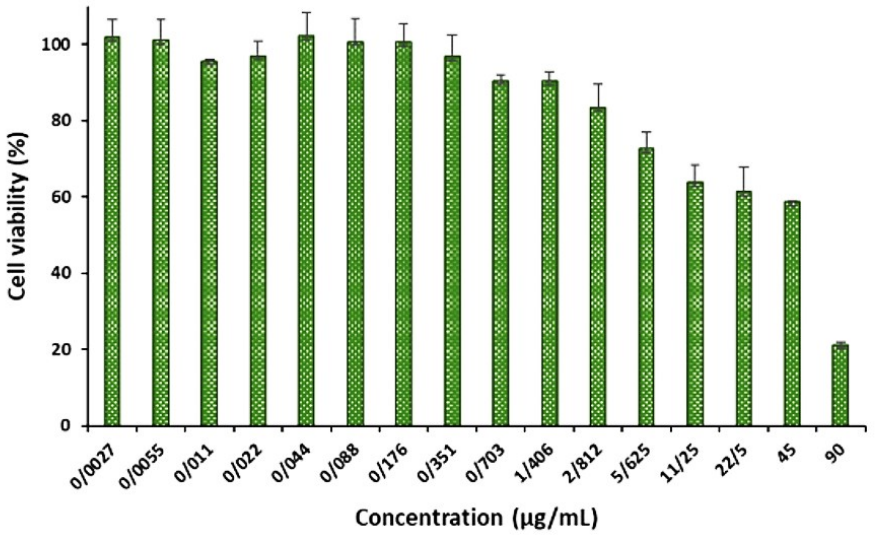

Figure 12-S: Conferone cytotoxicity on MDA-MB-231 cell lines, by MTT method.

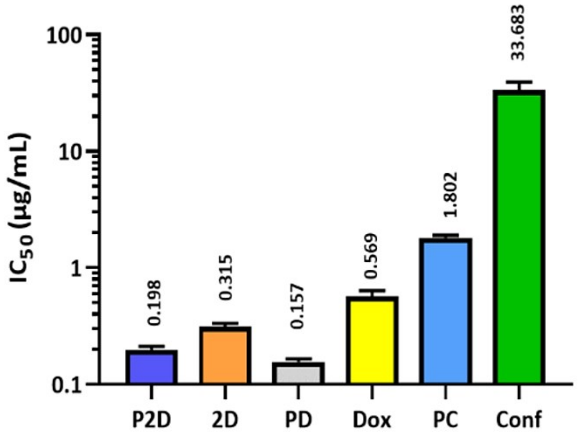

Figure 13-S: IC_{50} plot of all formulations, P2D, 2D, PD, Dox, PC and Conf; calculated by prism software ($P < 0.0001$).

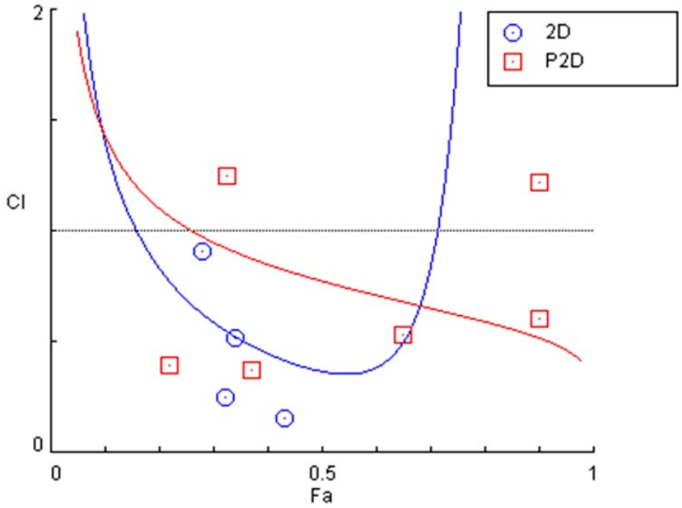

Figure 14-S: Combination Index (CI) plot, for P2D and 2D.

Table 2-S: Combination index (CI) results for P2D and 2D, in different doses, calculated by CompuSyn software.

**Total dose (ppm)**	**P2D**		**2D**	
	**Fa**	**CI**	**Fa**	**CI**
**0.058**	0.9	0.605	0.873	20.054
**0.117**	0.9	1.221	0.891	93.898
**0.234**	0.65	0.533	0.946	7739.77
**0.468**	0.37	0.370	0.431	0.156
**0.937**	0.221	0.393	0.324	0.249
**1.875**	0.326	1.25	0.341	0.518
**3.75**	0.284	2.101	0.282	0.909

Table 3-S: Cell cycle results of formulation on MDA-MB 231 cell line using flowcytometery.

**formulations**	**Cell cycles (%)**			
	**Sub G_0**	**G_0/G_1**	**S**	**G_2/M**
**P2D**	12.84	20.75	23.60	33.63
**2D**	15.63	53.88	17.91	11.25
**PD**	12.71	10.73	23.82	37.29
**Dox**	9.07	7.07	10.09	32.32
**PC**	1.29	10.03	8.05	38.93
**Conf**	3.61	59.84	18.27	16.40
**P**	3.78	46.31	11.45	29.83
**Control**	1.37	55.46	17.67	24.24

Table 4-S: Results of apoptosis of MDA-MB-231 cell line treated with formulation (P2D, 2D, PD, Dox, PC and Conf) using flowcytometery

**Cell %**	**Viable**	**Early apoptosis**	**Late apoptosis**	**Necrosis**
**P2D**	0.95	1.45	93.9	3.72
**2D**	12.2	22.6	36.1	29.2
**PD**	7.17	4.89	74.9	13.0
**Dox**	51.7	17.9	8.27	22.1
**PC**	45.4	12.0	37.8	4.77
**Conf**	81.4	3.97	14.1	0.55
**Control**	99.5	0.0	0.0	0.50

Table 5-S: Western blotting results of P2D, fold change of Bax, Bcl2, pro-Casp9, Cleaved-Casp9, pro-Casp3, Cleaved-Casp3, pro-Casp7, Cleaved-Casp7 and P27 proteins related to control (= 1).

**Fold change**	**proteins**								
	**Bcl-2**	**Bax**	**Pro-casp9**	**Cl-casp9**	**Pro-casp3**	**Cl-casp3**	**Pro-casp7**	**Cl-casp7**	**P27**
**P2D**	0.839	1.7	0.634	1.76	0.89	1.82	0.696	1.033	1.04
